# From Screening to Optimization: Strategic Implementation of Design of Experiments (DOE) for Robust Nanoparticle Formulation

**DOI:** 10.3390/polym18162034

**Published:** 2026-08-21

**Authors:** Ritu Gupta, Mahua Sarkar, Huan Xie

**Affiliations:** College of Pharmacy and Health Sciences, Texas Southern University, 3100 Cleburne St, Houston, TX 77004, USA; connect2rgupta@gmail.com (R.G.); mahua.sarkar@tsu.edu (M.S.)

**Keywords:** nanoparticle, optimization, quality by design (QbD), response surface methodologies (RSM), central composite design (CCD), Taguchi, AI/ML, nanomedicine, Design-Expert

## Abstract

Design of experiments (DOE) offers a powerful, systematic framework for optimizing nanoparticle (NP) formulations by replacing inefficient one-factor-at-a-time (OFAT) methods. By enabling the simultaneous evaluation of multiple variables, DOE uncovers critical factor interactions and identifies true global optima—critical for quality-by-design approaches. Despite its potential for systematic innovation, DOE remains underutilized in nanomedicine due to its perceived complexity; this review provides a practical roadmap to bridge the gap between statistical theory and robust NP optimization. It provides a practical overview of DOE concepts, including factor selection, design choice, graphical interpretation of results (perturbation/contour plots), model validation (regression analysis and ANOVA), and numerical optimization via desirability function (D). Common pitfalls and best-practice strategies are discussed to support reliable model building and decision-making. A practical case study on poly(lactic-co-glycolic acid) (PLGA) NPs illustrates a multistage workflow: utilizing Taguchi screening to isolate key factors, followed by central composite design (CCD), for precise surface mapping. Numerical optimization using Design-Expert^®^ software maximized EE% (highest importance) within size/zeta ranges, yielding optimal conditions (5 mg drug amount, 4 mL aqueous volume; D = 0.961). Confirmation runs (EE 41.2%, NP size 124 nm, zeta potential −15 mV) validated predictions (EE 47.6%, NP size 133 nm, zeta potential −17.2 mV), confirming model reliability. Ultimately, by bridging conceptual foundations with practical implementation, this review aims to encourage broader adoption of DOE, particularly among emerging formulation scientists, and serves as a roadmap to accelerate scalable NP development, fostering data-driven innovation and improving efficiency in nanomedicine research. Moreover, future integration of artificial intelligence (AI) and artificial neural networks (ANNs) with DOE will drive a predictive, data-driven approach to NP optimization—accelerating robust, scalable, and regulatory-ready nanomedicine development with fewer experiments.

## 1. Introduction

Pioneered by Fisher, Taguchi, Box, and Deming, design of experiments (DOE) combines mathematical models, statistical tools, and graphical optimization to thoroughly understand factor–response relationship [1,2,3,4,5,6,7,8,9].DOE enables systematic optimization of the system (product/process) by efficiently exploring experimental space and detecting interactions with minimal runs, in contrast to the traditional one-factor-at-a-time (OFAT) approach. By establishing a robust design space, it optimizes resource utilization, aligning built-in quality with sustainable and scalable manufacturing [10,11] across diverse industrial sectors, including construction [12], 3D printing [13], agriculture [14], food [15,16], chemistry [17,18], and pharmaceuticals [19,20].

DOE is a core statistical tool within quality by design (QbD), enabling systematic process understanding, identification, and optimization of critical factors to ensure quality is built into pharmaceutical processes from the start. DOE supports QbD by identifying critical quality attributes (CQAs) defined in the quality target product profile (QTPP) for a robust, regulatory-compliant product [21]. QbD is a proactive engineering strategy that ensures process performance and product quality are built in from the start, rather than tested at the end [22,23]. QbD is a holistic approach to manufacturing that utilizes statistical, mathematical, and risk management tools to define a multidimensional design space by linking critical quality attributes (CQAs) to critical process parameters (CPPs) and critical material attributes (CMAs) for consistent product/process performance. QTPP is a “prospective summary of quality characteristics” of the final drug product to ensure desired quality, safety, and efficacy (e.g., stable, injectable nanocarriers with an extended-release profile and superior therapeutic effect) [24]. CQA is a physical, chemical, biological, or microbiological property maintained within limits to meet the QTPP standards and ensure product quality [25]. While measured responses provide raw data, CQAs provide pharmaceutical context; for instance, entrapment efficiency (EE%) validates CQA of drug loading, while size and zeta potential define CQA of biodistribution and stability. CMAs (e.g., drug amount and solvent volume) and CPPs (e.g., infusion rate and mixing speed) are variables or factors manipulated to study their impact on the CQA [26].

QbD integrates DOE into a systematic, risk-based framework, extending its application beyond formulation optimization. DOE designs are used to identify critical material attributes (CMAs) and critical process parameters (CPPs) that influence NP quality. Compared with empirical methods, integrating QbD with DOE enhances formulation consistency, process understanding, scalability, and regulatory compliance. For instance, CCD and BBD designs are widely used to optimize self-emulsifying drug delivery systems (SEDDS) by efficiently identifying critical variables, interactions, and robust formulation design spaces for lipid-based formulations [27]. DOE-based QbD optimization of transferrin-conjugated SPIONs (superparamagnetic iron oxide nanoparticles) identified critical variables, optimized quality attributes, and supported development of targeted neuroprotective nanoplatforms for Alzheimer’s disease management [28]. In another study, DOE optimizes electrospinning parameters for polycaprolactone (PCL) scaffolds for tissue-engineered blood vessels by controlling fiber morphology and mechanical properties [29]. In a different investigation, bortezomib-loaded PLGA nanobubbles were optimized using BBD by evaluating drug, polymer, and stabilizer variables for sustained release in prostate cancer treatment [30]. QbD-guided CCD optimized risedronate-loaded cubosomes by evaluating formulation variables, achieving improved NP properties, skin permeation, and transdermal bioavailability compared with marketed oral tablets [31]. In a recent study, BBD optimized inhalable isoniazid–pyridoxine hydrochloride (INH–PDX) nano-embedded microparticles for tuberculosis treatment. It identified critical parameters to achieve optimal aerosol properties and enhanced lung delivery while mitigating neuropathy risks [32]. Further research demonstrates that DOE serves as a QbD tool to optimize lipid nanoparticles (solid lipid nanoparticles—SLN and nanostructured lipid carriers—NLC) by evaluating critical variables affecting NP quality attributes for nose-to-brain delivery [33]. DOE optimized budesonide-loaded Eudragit S-100 nanosponges by evaluating formulation variables, improving drug entrapment, release, and therapeutic efficacy in inflammatory bowel disease [34]. In a further study, DOE was used to compare top-down (wet milling, high-pressure homogenization) and bottom-up (chemical precipitation) approaches for meloxicam nanocrystal production. It revealed distinct process mechanisms, effects on nanocrystal properties, downstream processing impacts, and dissolution performance differences [35]. DOE-guided formulation optimization of epigallocatechin-3-gallate (EGCG)-loaded nanophytosomes (phospholipid-drug complex) improved their physicochemical properties and enhanced in vivo anti-inflammatory performance [36]. QbD-driven DOE optimized an *Ipomoea cairica*-derived silver nanoparticle nanogels, improving stability, release, skin permeation, and anti-inflammatory performance [37]. Another investigation highlighted a 3^2^ factorial design and response surface methodology to optimize cucurbitacin B-loaded core–shell lipid–polymer hybrid NPs, mapping excipient ratios to achieve controlled particle size and high entrapment efficiency [38].

Yingngam et al. (2025) reviewed the use of DOE for efficient pharmaceutical process optimization and systematic scale-up [39]. However, a recent survey by the European Compliance Academy (ECA) highlights underutilization of DOE in pharmaceutical manufacturing processes. Out of 51 participants, 65% use DOE at least “sometimes,” while only 6% use it daily. Despite 57% of respondents representing large firms (>500 employees), nearly 30% reported using DOE rarely or not at all [40]. Subsequent ECA survey (2025) revealed that 32% of participants identified barriers to DOE adoption, including a lack of experience/technical support, high software costs, management resistance, unavailable production lines, approval format uncertainty, time consumption, and perceived complexity in GMP environments [41]. Another literature review identified 16 barriers limiting widespread DOE application. The key obstacles were categorized into three distinct barrier groups: business (e.g., resistance to change and weak managerial support), educational (e.g., poor statistical background and inadequate training), and technical (e.g., complex methodology, insufficient software, and resources) [42]. Martín Tanco (2010) surveyed 101 participants from academics, consultants, and practitioners interested in DOE [43]. Survey results showed that low managerial commitment and weak statistical knowledge were the most significant obstacles.

**Figure 1:**
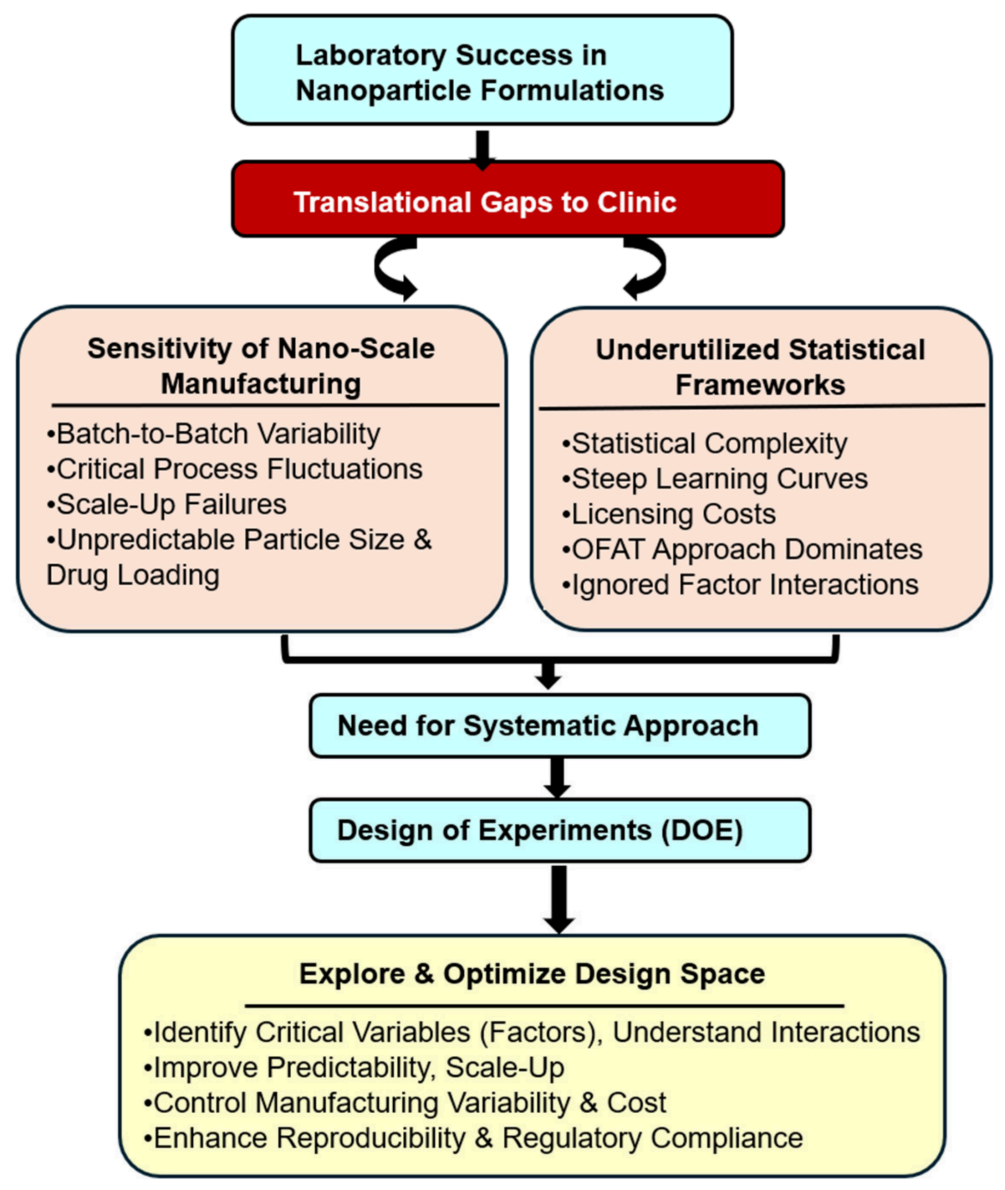
Nanoparticle formulation challenges and design of experiment (DOE) to address them.

In the same context, despite its significant potential for optimizing NP formulations, DOE remains underutilized due to methodological complexity, steep learning curves, software costs, and continued reliance on one-factor-at-a-time (OFAT) approaches. Additionally, challenges in selecting appropriate experimental designs and interpreting complex datasets further limit its broader adoption in NP development (Figure 1). In addition, nanoscale manufacturing presents unique challenges, including numerous formulation variables, complex factor interactions, sensitivity to process fluctuations, batch-to-batch variability, high characterization costs, and unpredictable NP size and drug loading. Together with the underutilization of statistical DOE frameworks, these factors continue to hinder reproducible formulation development and scale-up. Bridging the translational gap between laboratory NP success and clinical application requires a highly systematic approach. Implementing DOE addresses these hurdles by mapping the design space, identifying critical factor interactions, controlling batch variability, and ultimately enhancing regulatory compliance.

**Figure 2:**
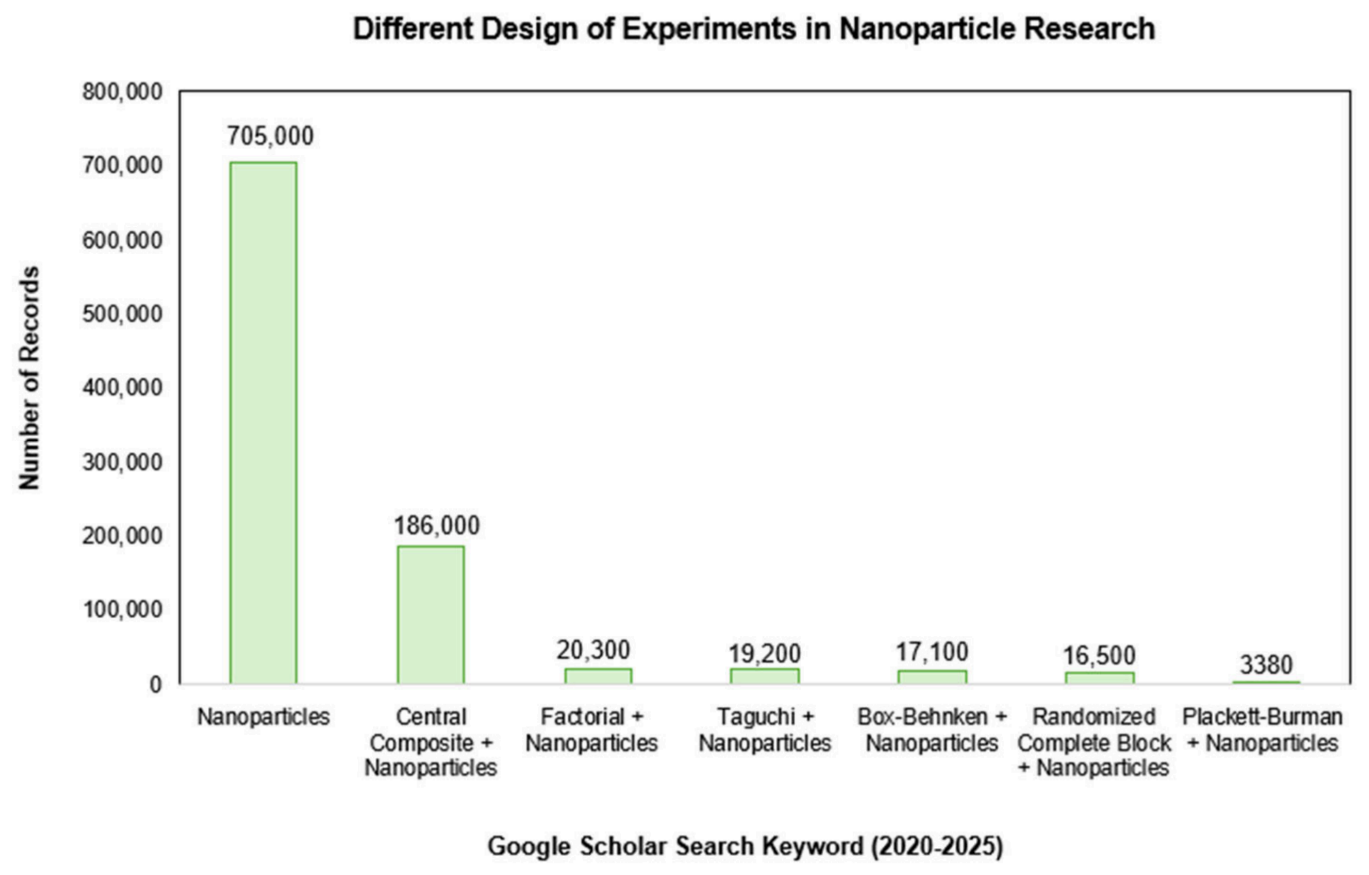
Underutilization of Design of Experiments (DOE) in Nanoparticle Research: Google Scholar Analysis, 2020—2025. The search was conducted using different DOE designs keywords (central composite, factorial, Taguchi, Box—Behnken, randomized complete block, and Plackett–Burman).

A Google Scholar search (2020—2025) for “nanoparticles” yielded ~705,000 publications; however, including specific DOE methods sharply reduced results, with central composite design (~186,000 studies) most common, highlighting continued underutilization of systematic DOE-based optimization in NP research (Figure 2).

This review presents a poly(lactic-co-glycolic acid) (PLGA) NPs case study, developed by the authors, to demonstrate the practical application of DOE in pharmaceutical formulation development. NP formulations are promising drug delivery platforms that can enhance drug solubility, targeting, and controlled drug release [44,45,46] but face manufacturing variability due to a lack of systematic optimization approaches. The case study presented in this manuscript is entirely the authors’ original research work. It details experimental objectives, factor selection, statistical analysis, graphical interpretation, model validation, and numerical optimization using Design-Expert^®^ software(version 13). While Design-Expert^®^ software was used for experimental design, analysis, and optimization, this does not imply its promotion or endorsement. Equivalent analyses could be performed using other commercial packages (Minitab, JMP, and SigmaXL) or open-source environments (R and Python).

Overall, this article provides a practical overview of DOE concepts to guide researchers—especially early-career scientists—in translating DOE outputs into robust, data-driven NP formulation optimization.

## 2. Design of Experiments (DOE) Workflow and Fundamental Principles

### 2.1. Design of Experiments (DOE) Workflow

**Figure 3:**
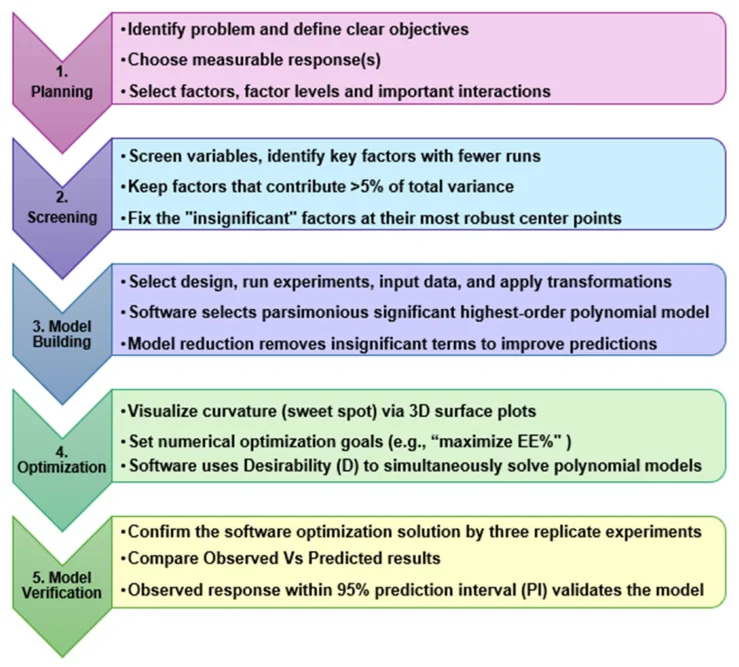
General five-step workflow for design of experiments (DOE).

The general workflow of design of experiments (DOE) is schematically presented in Figure 3 for clarity. This five-stage process systematically transitions from defining objectives, responses, and factors to screening variables and retaining influential factors with larger effects or higher variance contributions (in the ANOVA table) for further optimization [47] and then optimization of retained factors using quadratic models and 3D response surfaces. Finally, the desirability function identifies the global optimum, which is confirmed through experimental replicates. Observed responses within the 95% prediction interval validate the model and confirm its ability to predict new data.

This section focuses on the planning phase, including defining the problem and objectives, identifying factors and their levels, selecting measurable responses, designing resolution, and addressing confounding. Model development, optimization, and validation are discussed in later sections.

#### 2.1.1. Defining the Problem and Specific Objectives

A clear and succinct problem statement is necessary to better understand what needs to be done. Once the overall aim of the experiment is established, the experimenter needs to define specific, measurable, attainable, relevant, and time-bound (SMART) objectives [48]. Some common goals/objectives for conducting an experiment include screening, optimization, confirmation, discovery, and robustness testing [49].

#### 2.1.2. Identifying Factors and Factor Levels

**Figure 4:**
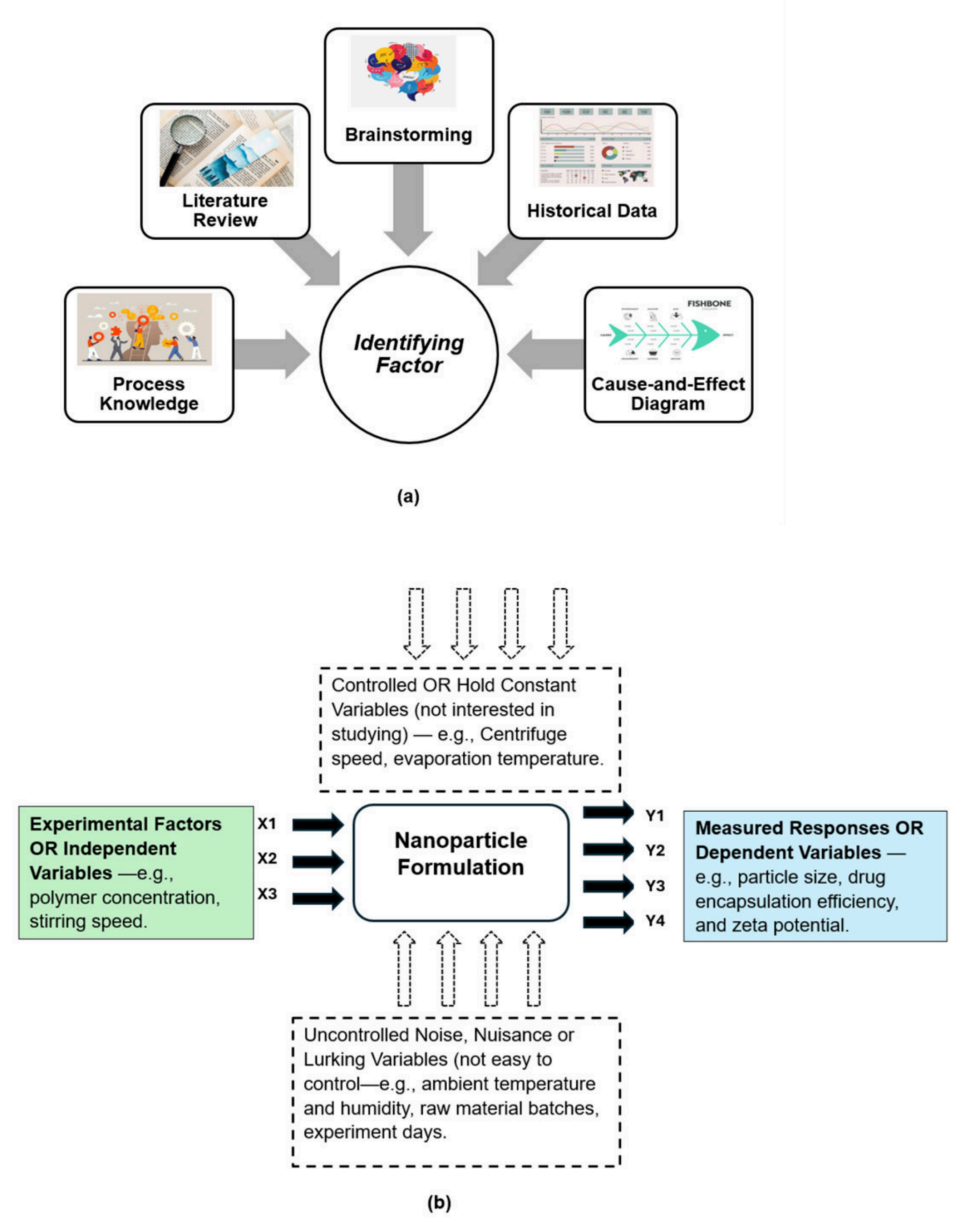
(**a**) Comprehensive factor identification methods used during the initial planning phase of design of experiments (DOE). (**b**) Classification of different factors.

Factors (CMAs and CPPs) are variables intentionally varied in experiments to observe their impact on outcomes. In NP formulations, examples include solvent type, stirring speed, and drug-to-polymer ratio. All factors need to be “mutually independent,” ensuring that a change in one factor does not cause a change in another [50]. DOE begins by identifying potential factors through literature, process knowledge, past data, brainstorming, or cause-and-effect (Ishikawa or Fishbone) diagrams [26] (Figure 4a). These factors are then classified into experimental, controlled (held constant), or uncontrolled (nuisance/noise) variables [51] (Figure 4b).

**Figure 5:**
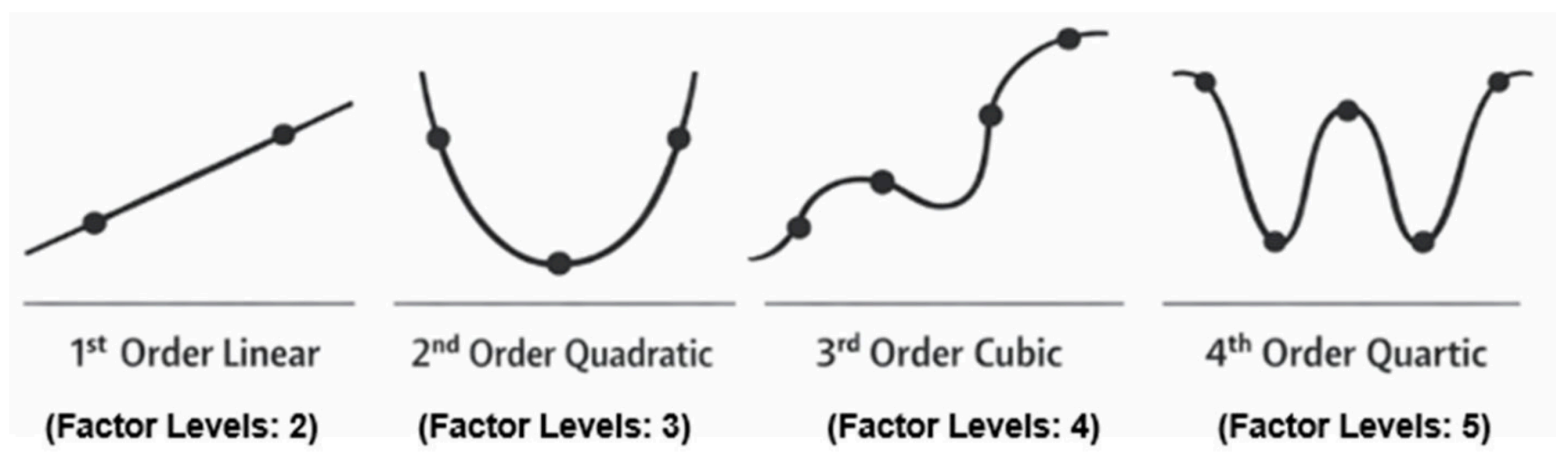
Polynomial orders required to model numeric factor-response curves and corresponding minimum factor levels needed.

Once experimental factors are selected, specific factor levels must be defined. Factor levels represent specific variable settings and are chosen based on study objectives, process complexity, and available resources [50]. While quantitative factors (e.g., drug amount) often start with two levels (low/high), qualitative factors (i.e., polymer type) need more [52]. Numeric factors often follow smooth curves with responses. Higher-order polynomials approximate these curved response shapes [53], as shown in Figure 5. More levels are needed for complex shapes, as listed in Table 1. To maintain reliable results in DOE, factor levels should be chosen carefully; too many levels reduce resolution and complicate higher-order analysis [50]. These levels are typically evenly spaced but not mandatory [54]. Closely spaced levels can miss effects in noise, and too-far-apart levels overextend into extreme nonlinear regions, yielding misleading RSM conclusions [55,56].

#### 2.1.3. Choosing Measurable Responses

Responses are key output variables (e.g., particle size and %EE)—preferably continuous—that reveal product/process performance [51]. Responses must be measurable using accurate, precise, robust, and validated methods to avoid measurement bias [52,57]. We also need to specify the instrument, operator, units, and decimal places to be used.

#### 2.1.4. Main Effects and Interactions

Understanding factor interactions is essential for accurate DOE interpretation [50]. Main effects describe the impact of one factor independently (e.g., stabilizer may reduce NP size irrespective of other factor settings), while interactions occur when a factor’s effect depends on another (non-additive) [58,59]. For instance, mixing speed may only influence NP size when paired with a specific solvent setting. The number of two-factor and three-factor interactions can be obtained by following Equation (1) and Equation (2), respectively [52,60]:

(1) $$N(2)=\frac{k\;(k-1)}{2}$$

(2) $$N(3)=\frac{k\left(k-1\right)(k-2)}{6}\;$$

where *k* is factor number and *N*(2) and *N*(3) are number of two-factor and three-factor interactions, respectively. For instance, four factors yield six two-factor interactions and four three-factor interactions. Experiment size depends on the factors studied and active interactions.

#### 2.1.5. Confounding and Design Resolution

Confounding occurs when factor effects overlap (with other factors/interactions) into single estimates, creating alias structures [61]. If Factor A is aliased with interaction AB, it is difficult to distinguish whether observed significance arises from Factor A or the overlapping interaction effect AB. Confounding can be addressed during the design phase via randomization, restriction, and matching. Randomization assigns treatments randomly. Restriction limits assignments to specific confounder levels, while matching ensures group comparability on confounders [62,63]. Design “resolution” determines the level of confounding of main effects and interactions [49,64,65].

- Resolution III: Main effects clear of other mains but aliased with two-factor interactions (2FI); two-factor interactions confound each other (e.g., 2^3−1^-fractional design).
- Resolution IV: Main effects clear of two-factor interactions (alias with 3FI), but two-factor interactions alias with each other (e.g., 2^4−1^-fractional design).
- Resolution V: Mains and two-factor interactions are both clear of each other (alias with 4FI/3FI) (e.g., 2^5−1^-fractional design).

### 2.2. Fundamental Principles of DOE

Before discussing specific experimental designs, it is important to recognize that all structured experimentation is built on three foundational principles. The following section explores replication, randomization, and blocking—the core principles that isolate true factor effects, minimize environmental noise, and guarantee statistical validity.

#### 2.2.1. Replication

Replication involves repetitions of full or partial experiments under various conditions. It estimates “pure error,” that is, the response variability unexplained by experimental factors. Replication improves signal-to-noise ratio, boosts experimental power, and reduces standard errors to detect smaller treatment effects, even when uncontrollable noise exists [66]. However, since each experiment costs time and resources, there is a trade-off between accuracy and budget [67].

#### 2.2.2. Randomization

Randomization is the process of randomly assigning experimental runs or factor combinations to minimize the effects of uncontrolled and unknown lurking variables (i.e., ambient temperature, operator handling, or equipment differences). It prevents systematic bias, spreads noise across runs, reveals long-term variability, and ensures observed effects result from the tested factors, not external influences [68].

#### 2.2.3. Blocking

Blocking increases experimental precision by removing the effect of known nuisance variables (e.g., raw material batches, experimental days/shifts, and machines) [69]. In blocking, similar experimental units are grouped into blocks; then treatments are randomly assigned within blocks, for example, grouping experiments by raw material batches [67]. This approach balances variations across runs, minimizing their impact and enabling more accurate evaluation of the experimental factors studied [67,69].

## 3. Overview of Design of Experiments (DOE)

Design of experiments (DOE) represents the entire strategy for solving a problem, including defining the objective, identifying critical process variables, conducting experiments, and analyzing data to optimize the system, whereas DOE methodologies—such as Plackett–Burman designs (PBD), Taguchi arrays, and response surface methodology (RSM)—are the specific structured statistical tools selected during the planning phase to execute that strategy and achieve distinct research objectives. The selected DOE methodology determines how the experiment is structured, including the number of runs, factor combinations, and analytical approach, based on the experimental objective; for example, PBD rapidly screen multiple variables in minimal runs, Taguchi designs build process robustness against uncontrollable noise, and RSM fine-tunes critical factors to pinpoint exact mathematical optima. The distinction can be understood through the following two examples.

For example, if a baker follows the full DOE framework to develop a better cake recipe, they start by establishing their goals, selecting factors like sugar and oven temperature, choosing a statistical plan, running trials, and analyzing the results. The specific DOE methodology (PBD, Taguchi, or RSM) chosen within that framework dictates how those trials are run: using a PBD allows them to screen through many ingredients quickly in a few runs, applying Taguchi design ensures the recipe remains consistent even if oven temperatures fluctuate, and switching to a RSM (like CCD and BBD) helps fine-tune the exact temperature and baking time for optimal fluffiness. Similarly, if a researcher follows the full DOE framework to develop a lipid NP for RNA delivery, they start by establishing critical quality attributes; selecting factors like lipid ratio, sonication time, and pH; choosing a statistical plan; running formulation trials; and analyzing particle size and entrapment efficiency. The specific methodology chosen within that framework (chosen during planning phase) dictates how those trials are structured: using a PBD allows them to screen through eight different process parameters quickly in a few runs, applying Taguchi design ensures the formulation stays stable despite minor batch-to-batch manufacturing variations, and switching to an RSM helps pinpoint the precise lipid-to-drug ratio needed for maximum encapsulation efficiency.

### 3.1. DOE vs. One-Factor-At-a-Time

One-factor-at-a-time (OFAT) or one-variable-at-a-time (OVAT) approach systematically varies a single factor to observe its effect on the response [70], while trial-and-error experiments test arbitrary factor settings. Both methods miss interactions and true optima, wasting resources on redundant runs and failing to reveal cause-and-effect relationships. DOE overcomes OFAT limitations by using fewer runs, testing robustness, reducing variability, supporting regulatory compliance, and guiding informed follow-up experiments, ultimately saving time and resources [21,71].

### 3.2. Common DOE Types

Multiple DOE designs exist, and their goals often overlap rather than being mutually exclusive. Design choice depends on problem objective, number of factors and interactions, design complexity, statistical validity, desired robustness, ease of use, and cost/time constraints [67].

Screening designs are used in the early stage of experimentation to identify significant factors from a large set of variables, typically using simple linear models and limited interaction estimation. Examples include Plackett–Burman design (PBD), Taguchi, and fractional factorial designs. In contrast, response surface methodology (RSM) employs structured designs to develop detailed empirical models, capture factor interactions, and optimize the responses using higher-order (usually quadratic) models. Examples include three-level full factorial, central composite design (CCD), and Box–Behnken design (BBD). Optimal designs are flexible DOE methods used for screening or RSM, depending on experimental objectives and model needs. Full factorial designs serve as a benchmark for evaluating fractional factorial and optimal designs. Two- and three-level full factorial designs require $2^{k}$ and $3^{k}$ runs, respectively, where k is factor number. These test all possible combinations of factors at all levels to provide unbiased estimates of main effects and interactions without confounding or aliasing between effects. On the other hand, fractional factorial designs use a fraction of full factorial runs (1/2, 1/4, and 1/8), improving efficiency at the cost of resolution. Runs follow 2^k−*p*^ for two-level and 3^k−*p*^ for three-level fractional factorial designs, where k is factor number and *p* is fraction. Example: 2^5−1^ = 16 runs (half fraction of five factors) and 2^6−2^ = 16 runs (quarter fraction of six factors). A two-level full factorial is suitable for a few factors to fully understand interactions. A two-level factorial with added center points can detect whether the curvature exists, but it cannot estimate or model the curvature. Although a three-level full factorial can model curvature (technically an RSM), it is rarely used due to excessive runs. To map a response surface efficiently, scientists usually bypass full factorials and choose specialized fractional RSM designs that give the exact same curvature information with a fraction of the runs. Central composite design (CCD) starts with a simple 2^k^ factorial run and adds a few center and axial points, while Box–Behnken design is an efficient three-level fractional layout that completely avoids testing expensive or unsafe extreme combinations.

#### 3.2.1. Screening Design

Screening experiments are used to eliminate non-significant factors. They focus primarily on identifying significant main effects, leaving aside analysis of interaction effects. They are small, efficient, and exploratory designs used to boil down the number of key factors prior to conducting larger experiments for process optimization [72]. First-order screening designs estimate linear effects of factors on the response but cannot capture any bend or curvature in the response. Screening designs are typically Resolution III (e.g., Plackett–Burman, Taguchi, and fractional factorial designs), although Resolution IV designs, such as definitive screening designs (DSD), may be used when estimating main effects free from confounding with two-factor interactions is desired.

Plackett–Burman designs (PBDs) are highly efficient, non-factorial screening designs constructed as orthogonal arrays to estimate only the main effects. Run sizes increase in multiples of four (e.g., 12, 20, 24 runs) rather than geometric powers of two (ie, 2^k^) [73]. They can screen up to (*n*−1) factors in *n* runs. They are strictly Resolution III designs. Unlike classical fractional factorials, where a main effect is fully aliased with a specific two-factor interaction (e.g., A ≡ BC), in PBDs, each main effect is partially confounded across every two-factor interaction that does not involve that factor [74].

Taguchi methods are robust screening designs that minimize the effect of uncontrollable noise. Although often equivalent to fractional factorial designs, their primary objective is robust parameter design rather than interaction estimation. They use orthogonal arrays to minimize experimental runs. In doing so, they sacrifice interaction information and can screen up to (n−1) factors in n runs. Taguchi designs typically exhibit Resolution III–like aliasing when fully saturated (e.g., seven factors in an L_8_ array). However, their alias structure depends on the orthogonal array, column assignment, and defining relation, rather than the number of factors alone [75]. A defining relation is an algebraic equation (e.g., I = ABCD) that specifies how factors and interactions are confounded in a fractional factorial design. A Resolution III design has a defining relation with word length three, such as I = ABC, where main effects are confounded with two-factor interactions (A ≡ BC, B ≡ AC, and C ≡ AB). Correspondingly, a Resolution IV design has a defining relation with word length four, such as I = ABCD, where main effects are aliased only with three-factor interactions (A ≡ BCD, B ≡ ACD, C ≡ ABD, and D ≡ ABC), and two-factor interactions are aliased with other two-factor interactions (AB ≡ CD, AC ≡ BD, and AD ≡ BC) [60,76]. Fully saturated Taguchi arrays, such as the L_8_ array (eight runs) with seven two-level factors, exhibit Resolution III–like aliasing. Assigning fewer factors in above L_8_ array (e.g., four factors) reduces aliasing and allows limited interaction estimation but does not automatically convert it to a Resolution IV design. In contrast, the classical 2^(4−1)^ fractional factorial design (four factors, eight runs) is a true Resolution IV design with standard generator and defining relation.

The difference between Taguchi and fractional factorial designs can be illustrated using nanoparticle (NP) synthesis example. Both designs handle environmental variation or noise (e.g., room temperature and humidity) differently. A classical fractional factorial design identifies control factor settings (e.g., drug amount and infusion speed) that significantly change mean response (NP size and %EE). The unexplained variation is treated as residual error. Noise (e.g., room temperature and humidity) is kept constant (by strictly fixing temperature and humidity) or minimized through randomization and blocking. In contrast, Taguchi designs deliberately incorporate noise into the experiment (by conducting runs under different room temperatures and humidity levels) to identify control factor settings that make the response (size and %EE) insensitive to environmental variation. Thus, fractional factorial designs emphasize effect estimation by reducing noise, whereas Taguchi methods focus on robust parameter design under varying conditions. Although Taguchi designs are also used for screening, their primary objective is robustness rather than screening alone.

Introduced by Jones and Nachtsheim (2011), definitive screening designs (DSDs) are a modern class of three-level screening designs (using −1, 0, and +1 levels) that overcome the limitations of classical two-level designs like PBD or Resolution III fractional factorials [77]. They require a remarkably small number of runs—typically only 2k + 1 or 2k + 3 (where k is the number of factors)—making them highly cost-effective. Their unique mathematical structure ensures that main effects are completely clear. They are strictly orthogonal to one another and entirely uncorrelated with two-factor interactions or quadratic effects. Unlike traditional two-level designs that only map straight lines, DSDs include a center point (0) for factors. This allows them to identify nonlinear, curved relationships (quadratic effects) right during the initial screening phase, making them significantly more informative than classical screening designs [78].

In brief, PBDs can estimate only main effects with heavy interaction confounding, Taguchi designs provide limited interaction estimation with aliasing dependent on column assignment, while DSDs are less suitable when many interactions are important.

#### 3.2.2. Response Surface Methodology (RSM)

A first-order (linear) model shows simple up/down trends, while a second-order model captures curvature [79,80]. RSM relies on second-order designs—such as three-level full factorial, central composite design (CCD), and Box–Behnken design (BBD)—to estimate curvature using quadratic equations [64]. RSM models respond as functions of multiple factors, creating visual maps through 3D surfaces and contour plots. This enables simultaneous optimization of multiple factors while reducing the number of experiments, saving time, and lowering costs [81].

Central composite design (CCD) is a widely used RSM design for fitting second-order (quadratic) models when factor–response relationships are nonlinear. It consists of three types of design points with 2ᵏ + 2k + n runs, where k is factor and n is center points. Factorial points (2ᵏ) estimate linear effects and factor interactions using a full or fractional factorial. Axial (star) points (2k), placed at ±α, estimate quadratic (curvature) effects. Center points (n) estimate pure experimental error and assess lack of fit [82,83,84,85]. The inclusion of center points enables the estimation of pure experimental error and facilitates the assessment of curvature and lack of fit. However, adding center points does not convert a two-level design into a true three-level design; the underlying model remains primarily linear with an additional capability to detect nonlinearity. Typically, three to five center points are recommended for reliable estimation of pure error and curvature testing. Orthogonality is an important property in a CCD (preferred but not essential with modern computational tools). It ensures that main effects and quadratic terms are estimated independently. It minimizes correlation among factors (i.e., the effect of one factor can be calculated without being distorted by changes in another) and improves the accuracy and interpretability of regression coefficients. However, complete orthogonality of the design matrix depends on how the axial distance (alpha) and the number of center points are selected.

Box–Behnken design (BBD) is an independent quadratic RSM design without an embedded factorial core, using 2k(k − 1) + n runs, where k is factor and n is center points [86]. It requires at least three factors and avoids simultaneous extreme factor combinations by placing design points at the midpoints of cube edges. BBD efficiently estimates quadratic and interaction effects with fewer runs than CCD, making it ideal when extreme axial points are costly, impractical, or unsafe [87,88,89]. For three factors, 3^k^ full factorial design requires 27 runs; CCD typically requires 17 runs (8 factorial + 6 axial + 3 center points); BBD requires 15 runs (with three center points) for quadratic modeling efficiency.

#### 3.2.3. Optimal Designs

Optimal designs maximize statistical efficiency, particularly in constrained or irregular design spaces, by optimizing information-matrix criteria such as D- or I-optimality. Optimal designs are flexible DOE strategies that can be used for both screening and response surface methodology (RSM), depending on the experimental objective and model. While D-optimality minimizes the error of model coefficients (ideal for early-stage screening), I-optimality minimizes the error of the final predicted output (ideal for late-stage RSM optimization).

Several optimality criteria are used in pharmaceutical formulation development, including D-, I-, A-, G-, and E-optimality [90]. Among these, D- and I-optimality are most commonly used, while E-optimality is used less frequently. D-optimality criterion maximizes the “determinant of information matrix,” minimizing overall variance of parameter estimates (coefficients). This is ideal for screening [91,92]. I-optimality (IV-optimality or integrated variance optimality) criterion minimizes average prediction variance across the design space, improving prediction accuracy for new observations. It is commonly used in RSM when the primary objective is to develop predictive models rather than simply estimate coefficients [93,94,95]. A-optimality criterion minimizes the “trace of inverse information matrix,” minimizing average variance of individual parameter estimates. Unlike D-optimality, this A-optimality ignores covariances among coefficients [90,96]. G-Optimality minimizes the maximum prediction variance (i.e., worst-case prediction variance) across the design space, improving the worst-case prediction accuracy [97]. E-optimality criterion maximizes the “smallest eigenvalue of information matrix,” ensuring worst-case parameter variance is minimized. It protects against the worst-estimated parameter and improves parameter estimation stability [98]. In essence, G-optimality minimizes the worst prediction variance, whereas E-optimality minimizes the worst parameter variance.

Table 2 briefly outlines popular experimental designs, their strengths, limitations, and typical uses. It presents representative and widely used DOE designs rather than an exhaustive classification of all available experimental designs. To avoid excessive complexity and maintain the focus of the review, less commonly used or highly specialized designs were not included.

## 4. Statistical Analysis and Interpretation of DOE Results

After conducting the DOE experiment and collecting data, the next step is analyzing and interpreting the results to draw meaningful conclusions. This stage aims to identify factors impacting response and variability, determine their optimal levels, and assess opportunities for further improvement.

### 4.1. Model Selection & Hierarchy

Software hierarchically tests models (Mean → Linear → 2FI → Quadratic → Cubic), selecting the highest-order polynomial with significant and non-aliased terms, ensuring lower-order precursors are retained for any significant higher-order terms (e.g., keeping A if A^2^ is significant). The parsimony principle (also termed as Occam’s razor) favors the simplest adequate model, avoiding unnecessary complexity [152,153].

To balance accuracy and complexity, three selection methods are used for model refinement. Forward selection starts with a null model and adds significant terms one by one. Backward elimination starts with all terms and removes insignificant ones gradually [154]. The stepwise method combines both, with stricter entry criteria (*p* < 0.10) than removal (*p* > 0.15). Usually, quadratic models provide the ideal balance by capturing essential curvature to locate true optima, unlike oversimplified linear models or overfitted, noise-sensitive cubic models. A general polynomial model incorporating linear, interaction, and quadratic effects in the regression equation can be written as follows (Equation (3)):

(3) $$Y=\beta_{0}+\beta_{1}X_{1}+\beta_{2}X_{2}+\ldots +\beta_{k}X_{k}+\beta_{11}X_{1}^{2}+\beta_{22}X_{2}^{2}+\ldots \beta_{kk}X_{k}^{2}+\beta_{12}X_{1}X_{2}+\ldots +{\;\beta }_{k-1,k}X_{k-1}X_{k}+\varepsilon$$

Here, *Y* represents the predicted response, and *k* is the total number of factors. *X_1_* and X_2_ are individual factor (main effect) settings in coded units (−1, 0, or +1). The term *β_0_* is the intercept, a constant regardless of factor-level settings, and represents the overall mean response. When all factors are set at their center point level (coded value = 0), the value of the predicted response reduces to *β_0_*. The terms *β_1_* and *β*_2_ are constant coefficients (regressors) for linear effects; *β_11_* and *β_22_* are coefficients for quadratic effects; *β_12_* is a coefficient for two-factor interactions, respectively. Term ε represents the residual error associated with the model [118,155,156].

### 4.2. Model Robustness and Reproducibility

To ensure an RSM model is both robust (stable) and reliable (reproducible), statisticians look beyond simple fit and focus on predictive power and signal quality. The model validation step ensures statistical soundness and data fit before optimization (during the analysis phase). It checks ANOVA (*p*-values, lack of fit), R^2^ metrics (adjusted/predicted), and diagnostic plots (residuals normal plot, residuals vs. predicted) and confirms the mathematical equation adequately describes data [157].

#### 4.2.1. Regression Analysis (Adjusted and Predicted R^2^)

Regression models continuous variables for numerical prediction via R^2^ metrics or coefficient of determination, unlike ANOVA, which compares group means for categorical variables. Raw R^2^ statistic represents the proportion of variation explained by the model and is calculated as the ratio of the model sum of squares to the corrected total sum of squares (R^2^ = SS_model_/SS_CorTotal_). A value near 1 (R^2^ ~ 1) indicates strong explanatory power, and it is susceptible to “overfitting,” as it rises deceptively with every added term, regardless of statistical significance [152].

Adjusted R^2^ penalizes model complexity, rising only when new terms truly improve fit. While adjusted R^2^ assesses current dataset fit, predicted R^2^ evaluates performance on new data. Predicted R^2^ is preferred for model validation, especially for processes with hard-to-measure responses and noisy data (noisy processes can yield low R^2^ despite good models) [157]. For a robust model, the gap between adjusted and predicted R^2^ should be <0.2 to avoid overfitting. These metrics guide model reduction and prevent overfitting. Regression allows data transformation to stabilize non-constant (heteroscedastic) variance and enhance statistical power [158,159], but cannot represent true categorical variables numerically. Its coefficients ultimately enable prediction and navigation of the continuous design space toward optimal conditions.

#### 4.2.2. Adequate Precision

Adequate precision measures the signal-to-noise ratio in Design-Expert^®^ by comparing the predicted range (difference between maximum and minimum predicted response) to the average prediction error. Ratio > 4 indicates an adequate signal (model’s predicted response exceeds noise), enabling design space navigation [49].

#### 4.2.3. Coefficient of Variation (CV %)

The coefficient of variation quantifies internal precision by measuring experimental noise relative to the mean. A low CV (typically <15%) signifies superior consistency and reproducibility across runs.

#### 4.2.4. Prediction Error (PRESS or Predicted Residual Error Sum of Squares)

The PRESS statistic is a rigorous measure of a model’s predictive accuracy, evaluating the deviation between actual data points and forecasted values. A small PRESS value indicates high stability, ensuring individual data points do not disproportionately influence results. It is core to predicted R^2^: high PRESS yields low predicted R^2^, indicating an overfitting model [49]. Unlike standard R^2^ (which uses all data points for fit), PRESS uses leave-one-out cross-validation: remove one point, refit the model on the rest, predict the removed point, record the squared prediction errors (i.e., squared difference between the actual and predicted value), repeat the process for all points, and sum the results [152].

#### 4.2.5. ANOVA (F-Values, *p*-Values, and Sums of Squares)

ANOVA (analysis of variance) compares group means to identify significant factors and interactions for categorical variables (e.g., drug types/polymers). It is suited best for nonlinear relationships due to easy interpretability [153]. Key metrics in ANOVA include F-values, *p*-values, and sums of squares (SS). It partitions the total variability around mean (corrected total SS or SST) into model SS (explained) and residual SS (unexplained variability—including both lack of fit and pure error). A large residual SS reflects weaker factor effects and greater unexplained variability. The F-test measures significance against random error. The calculated F-ratio is compared with the critical value from statistical tables. If the F-ratio exceeds the critical value, it indicates that a factor significantly affects the response variable or that an interaction between factors is present [50]. While excellent for hypothesis testing and factor screening, ANOVA offers limited predictive power for continuous factor optimization compared to regression. In RSM, it serves as a robust validation tool before proceeding with optimization.

#### 4.2.6. Lack-of-Fit *p*-Value

The lack-of-fit test compares regression error (model inadequacy) against pure error (replicate variation) by comparing unexplained variation (lack-of-fit MS) with replicate variation (pure error MS). When these values are similar, F ≈ 1 indicates a non-significant lack of fit (*p* > 0.05) and a well-fitted model that adequately captures the true relationship. Adding center points allows for calculation of pure experimental error but does not make a two-level design three-level; the model remains linear, with an added check for nonlinearity [72,160]. Significant lack of fit signals the need for an RSM design to capture curvature.

#### 4.2.7. Common DOE Pitfalls and Avoidance Strategies

Careful attention to common pitfalls throughout DOE planning, execution, and analysis is essential for scientists optimizing a product/process. Vague objectives, hierarchy violations, or skipped validations produce unreliable models that fail regulatory scrutiny. Implementing rigorous strategies like hierarchical model building, center point replicates, and confirmation experiments guarantees robust, statistically sound predictive outcomes in RSM. Table 3 captures the frequent DOE pitfalls [72,161]—from poor planning to modeling flaws—and offers pharmaceutical scientists with actionable strategies to construct reliable, high-integrity RSM for NP formulation optimization.

### 4.3. Response Surface Interpretation

A response surface is a multidimensional mathematical and graphical model that maps the relationship between independent variables (factors) and a dependent response to identify optimal conditions and interaction effects [49].

#### 4.3.1. Design Space

**Figure 6:**
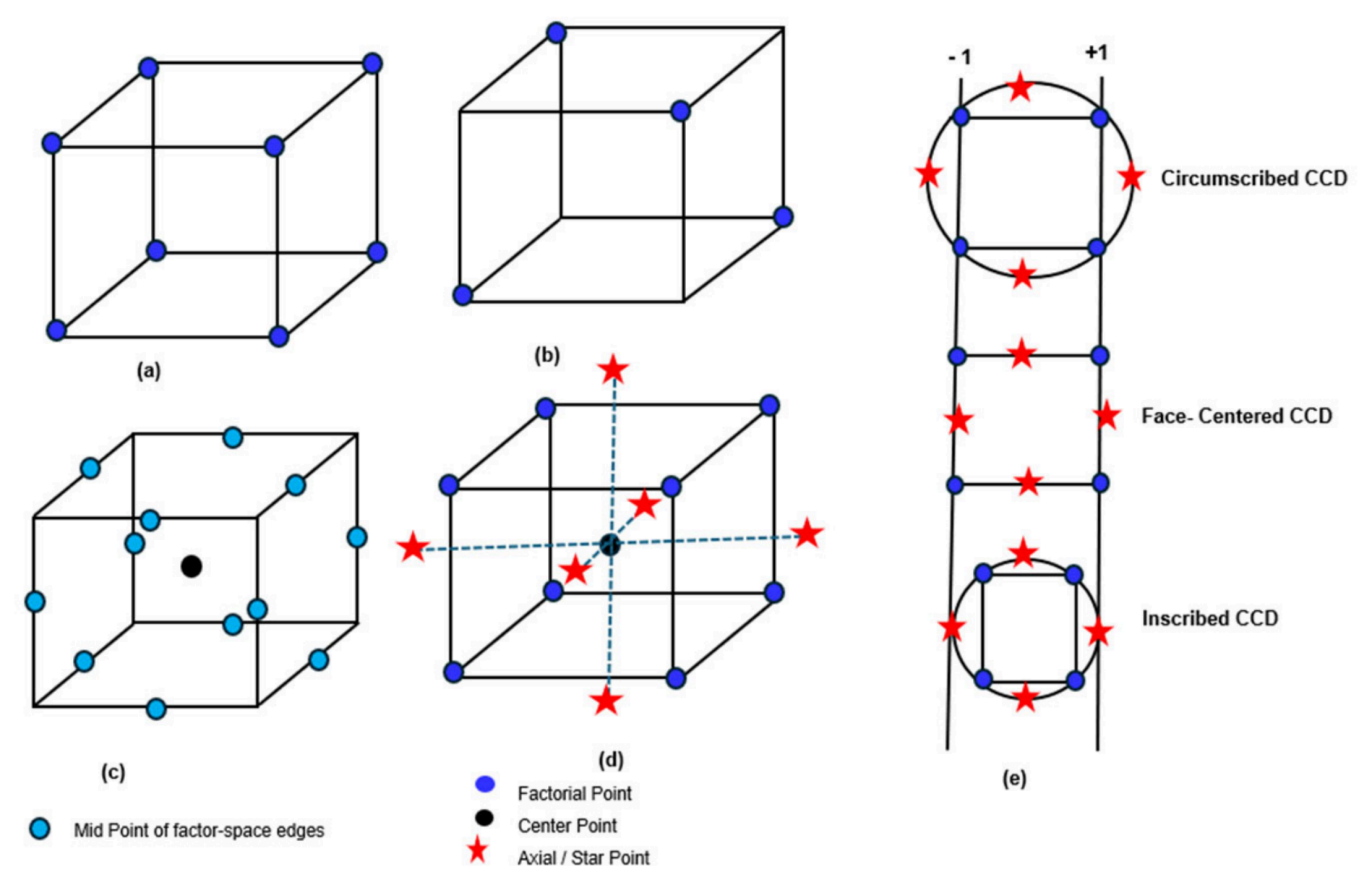
Common DOE designs: (**a**) 2^3^ full factorial design. (**b**) Fractional factorial design. (**c**) Box—Behnken design (BBD). (**d**) Central composite design (CCD). (**e**) Three varieties of CCD designs: (i) circumscribed CCD (or CCC); (ii) face–centered CCD (or CCF); and (iii) inscribed CCD (or CCI).

In RSM, the design space is the multidimensional zone defined by factor limits or boundaries (i.e., low-high), within which the model is constructed, estimated, and statistically valid for optimization [23,25]. In other words, design space serves as the valid “experimental territory” for optimization, typically visualized through 3D surface or 2D contour plots. Predictions are reliable within the design space (interpolation) but statistically risky outside it (extrapolation) due to unpredictable surface behavior. Depending on the design type (i.e., factorial or CCD), design space can be a cube or a sphere. Figure 6 illustrates geometries of common DOE designs.

#### 4.3.2. Design Stationary Points (3D Response Surface and 2D Contour Plots)

**Figure 7:**
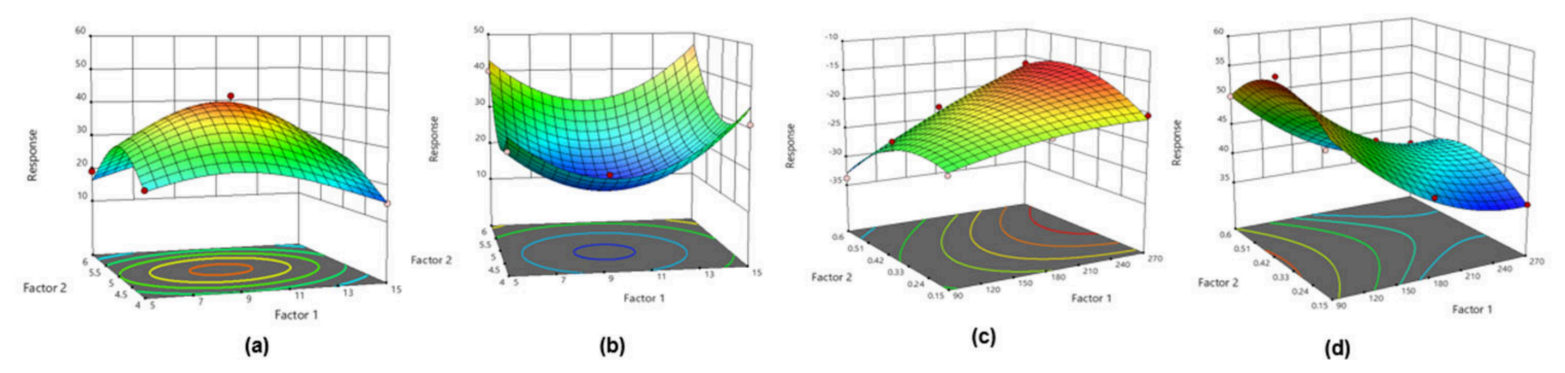
3D response surface plots illustrating various stationary points in RSM. (**a**) Peak or maximum with a downward curve. (**b**) Valley or minimum with upward curve. (**c**) Maximum outside the range. (**d**) Saddle point with mixed curvature (trade-off needed).

**Figure 8:**
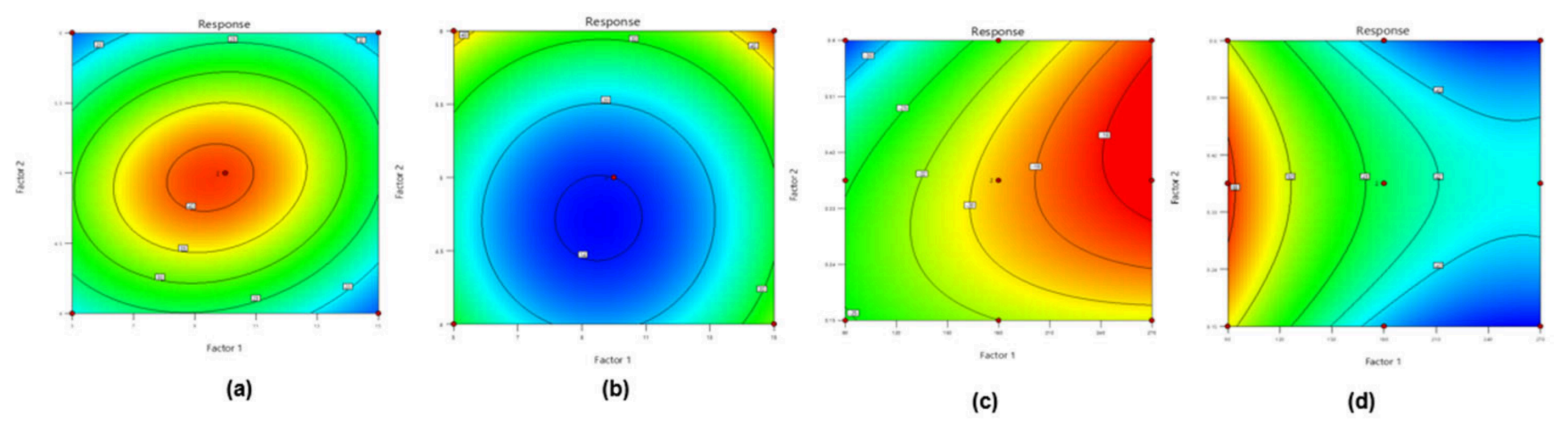
Various contour plots (2D) based on a polynomial quadratic model: (**a**) maximum; (**b**) minimum; (**c**) maximum outside the experimental region; and (**d**) saddle point.

In RSM, stationary points are key locations on the response surface that indicate optimal factor combinations for maximum (peak) or minimum (valley) response. They pinpoint critical “geographical solutions” where the rate of response change is zero (i.e., response slope is zero in all directions), defining stable design boundaries and factor sensitivity. Conversely, saddle points indicate complex curvature, suggesting that the true optimum may lie beyond the study’s experimental range [164,165,166]. Figure 7 and Figure 8 illustrate the primary types of stationary points encountered in RSM.

#### 4.3.3. Graphs for Model Interpretation

**Figure 9:**
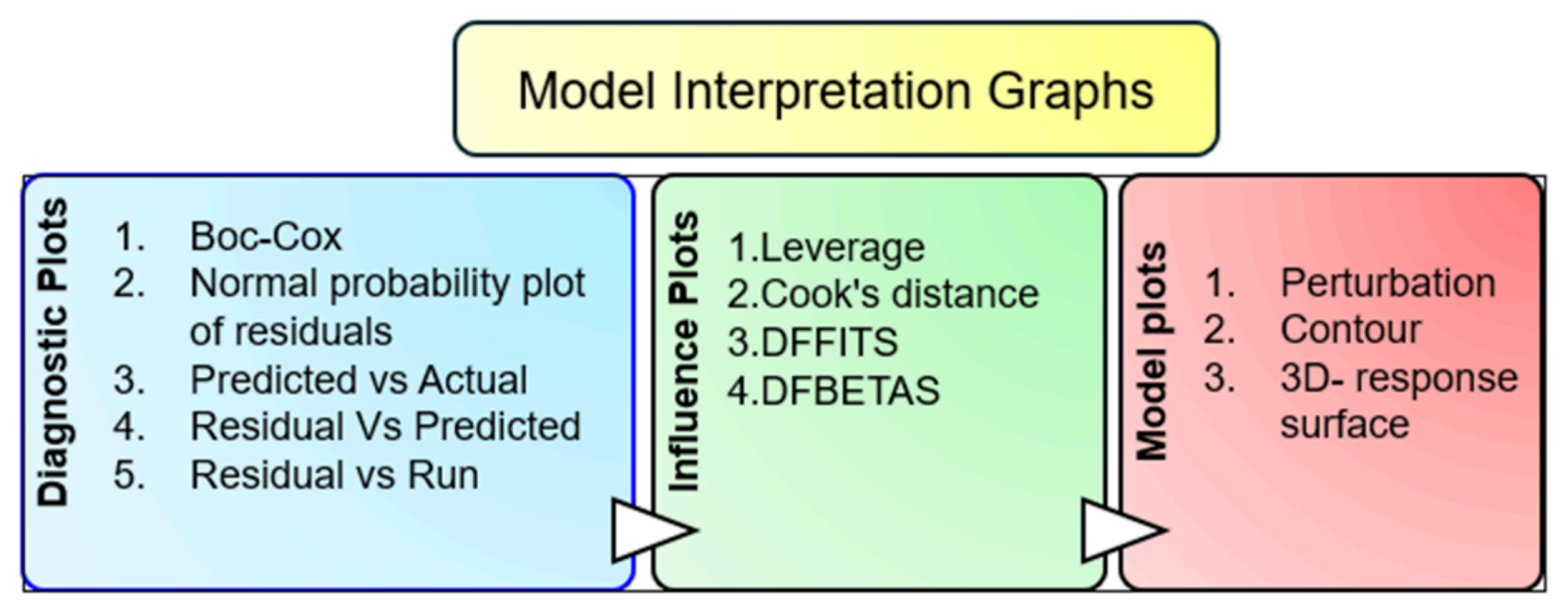
Types of graphs for model interpretation.

Design-Expert^®^ software offers a variety of graphs (Figure 9 and Table 4) to aid in the interpretation of the selected model. When reviewing the graphs, it is best to prioritize terms with significant effects. Diagnostic plots validate model assumptions and fit. Model graphs help in visualizing intricate data relationships and effects. Evaluation plots assess the impact of individual points on the model. They can flag high-impact points without necessarily implying poor fit [167].

#### 4.3.4. Mathematical (Numerical) Optimization & Desirability Function (D)

Design-Expert^®^ offers both graphical and numerical optimization. While graphical optimization suits single response, numerical (mathematical) optimization is superior for balancing multiple, potentially conflicting responses [171,172]. Numerical optimization utilizes the mathematical models derived from the ANOVA to search the entire experimental space for a “sweet spot.” Unlike graphical optimization (which uses visual overlays), numerical optimization uses a desirability function (D), a mathematical tool, not a statistical function, used to rank solutions by how closely they meet goals.

The desirability function approach, originally introduced by Derringer and Suich (1980), is the most widely used mathematical framework for multi-response optimization in design of experiments [171]. When evaluating a complex process (such as pharmaceutical formulations), multiple output responses (e.g., maximizing %EE and minimizing NP size) are simultaneously optimized. The desirability function solves this multi-objective dilemma by transforming several predicted response surfaces into a single, unified optimization metric (D) ranging from to 0 to 1. The first step in the method is converting each individual response (Y_i_) into a dimensionless individual desirability value (d_i_), where:

- d_i_ = 0: represents a completely unacceptable response value.
- d*_i_* = 1: represents the ideal or perfectly optimized response value.

**Figure 10:**
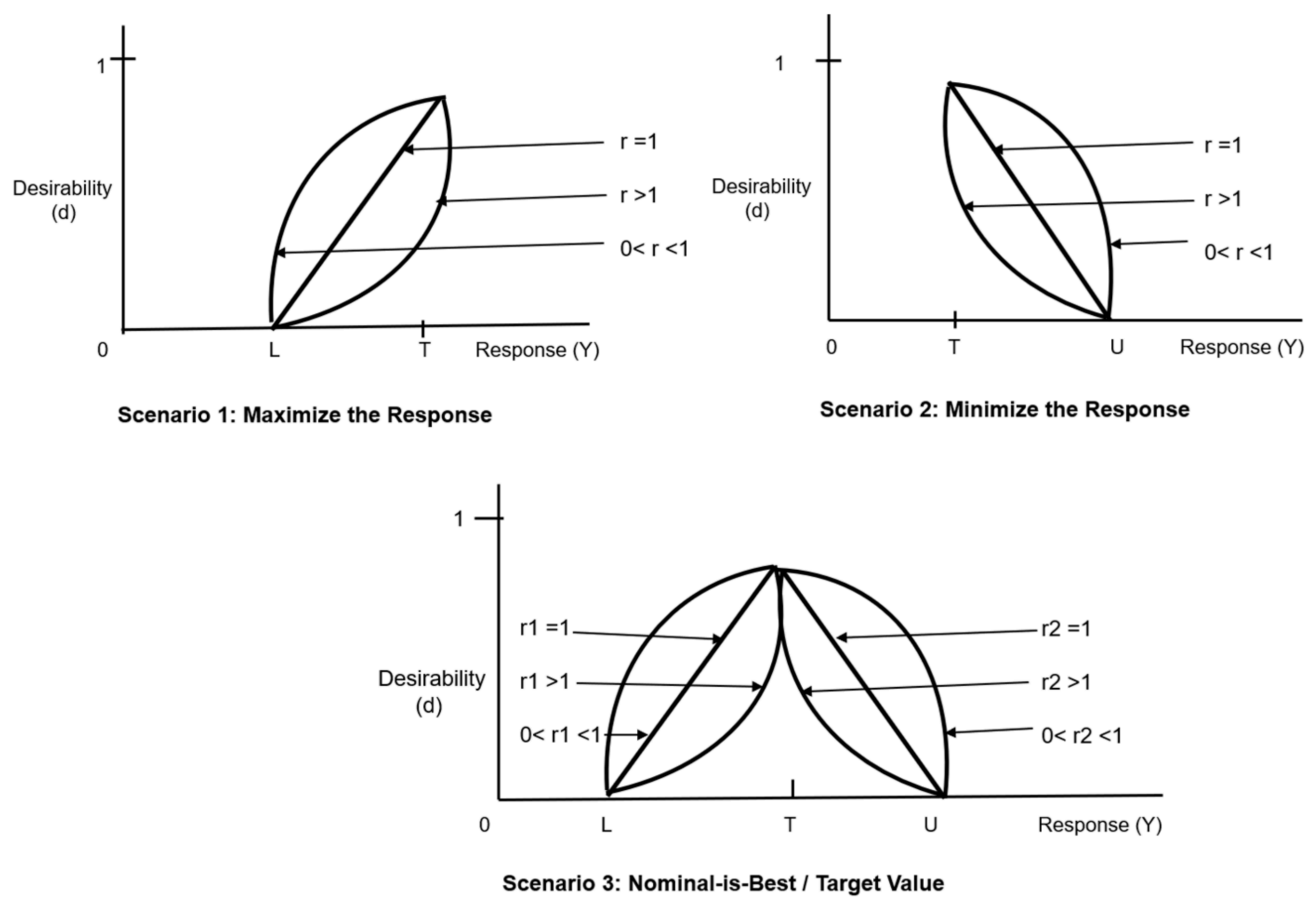
Individual desirability functions for simultaneous (multi-response) optimization.

Depending on the operational objective, the mathematical definition of desirability alters [49,90], as depicted in Figure 10.

**Scenario 1: Maximize the Response (e.g., %EE)**

When the objective is to maximize a response, a lower acceptable threshold (L) and a target maximum value (T) are specified.

(4) d={01(Y−LT−L)rY<LL≤Y≤TY>T

**Scenario 2: Minimize the Response (e.g., Particle Size)**

When the objective is to minimize a response, an upper acceptable threshold (U) and a minimum ideal value (T) are specified.

(5) d={10(U−YU−T)rY<TT≤Y≤UY>U

**Scenario 3: Nominal-is-Best/Target Value (e.g., pH)**

The two-sided desirability function assumes that the target value lies between the lower limit (L) and upper limit (U).

(6) $$d=\{\begin{array}{c} \begin{array}{c} 0\;{\left(\frac{Y-L}{T-L}\right)}^{r1}\\ 0\;{\left(\frac{U-Y}{U-T}\right)}^{r2} \end{array} \end{array}\;Y<L\;L\leq Y\leq T\;T\leq Y\leq U\;Y>U$$

The exponent “r” in the above equations is the weight that dictates how strictly the algorithm penalizes deviations from the ideal target.

- r = 1: linear desirability.
- r > 1: stronger emphasis on being close to the target.
- 0 < r < 1: less emphasis on closeness to the target.

These individual desirability values are then combined using the geometric mean D = (d1 × d2 × …dx)^1/x^, and an algorithm is applied to maximize this combined desirability function for simultaneous multi-response optimization [171]. The use of the geometric mean (multiplication) instead of the arithmetic mean (addition) is the key feature of this optimization approach. If any response does not satisfy its specification limits, its individual desirability becomes zero (di = 0). Since multiplying by zero yields zero, the overall desirability also becomes zero immediately. By using the geometric mean, the optimization scheme strictly penalizes any formulation that fails to meet quality or clinical targeting criteria (e.g., an unacceptable particle size), preventing a high %EE from masking a critical nano-size requirement. Design-Expert^®^ employs direct search methods to maximize the overall desirability function (D). While individual desirability functions are inherently non-differentiable, they can be replaced with differentiable polynomial approximations to enable the use of standard derivative-based optimization algorithms.

In Design-Expert^®^, users define goals (max/min/target/range) and importance weights (1 to 5 +) for individual responses. Based on the defined optimization goals, the software assigns an individual desirability score—ranging from 0 (unacceptable) to 1 (ideal)—to each response. These individual values are then consolidated into a single overall desirability using a geometric mean, and the software algorithm identifies the factor settings that maximize this value within the defined design space. The selected model is validated with “Confirmation runs,” either at optimal conditions or spread across the design space [173,174].

### 4.4. Model Verification (Post-Analysis)

Model verification (a post-DOE experiment step) uses new data, not used in model-building, to assess model adequacy and predict its future performance. It is recommended to confirm the model with multiple observations, preferably three to four runs at or close to the factor settings suggested by the numerical optimization—acknowledging that the mathematical optimum may be physically unattainable or economically impractical [49]. In model confirmation, observed responses are compared with Design-Expert’s prediction interval. If the observed response at the predicted optimum falls within the prediction interval (95% or selected PI), the model is considered validated [175]. If confirmation runs fall outside the prediction interval, it suggests model inadequacy, requiring a higher-order model or indicating that a local optimum was mistaken for the global optimum [49].

In case of blocked designs that group homogeneous units to control for known nuisance factors (e.g., raw material batches, days, or machines), multiple confirmatory runs across repeatable blocks should be performed to average out block effects. If restricted to one run, the lowest-effect block (with low SS in ANOVA, indicating minimal impact from specific experimental conditions) should be selected for the confirmation run. When post-experiment confirmation is impractical due to long duration/follow-up times (e.g., drug expiry), expensive equipment, or one-shot experiments (e.g., production line availability), “concurrent confirmation runs” can be built into the initial design [157].

We usually skip model verification (post-analysis step) when the objective is factor screening, especially if the process is non-critical or if physical and budgetary constraints prevent further confirmatory runs [157].

## 5. Case Study

**Figure 11:**
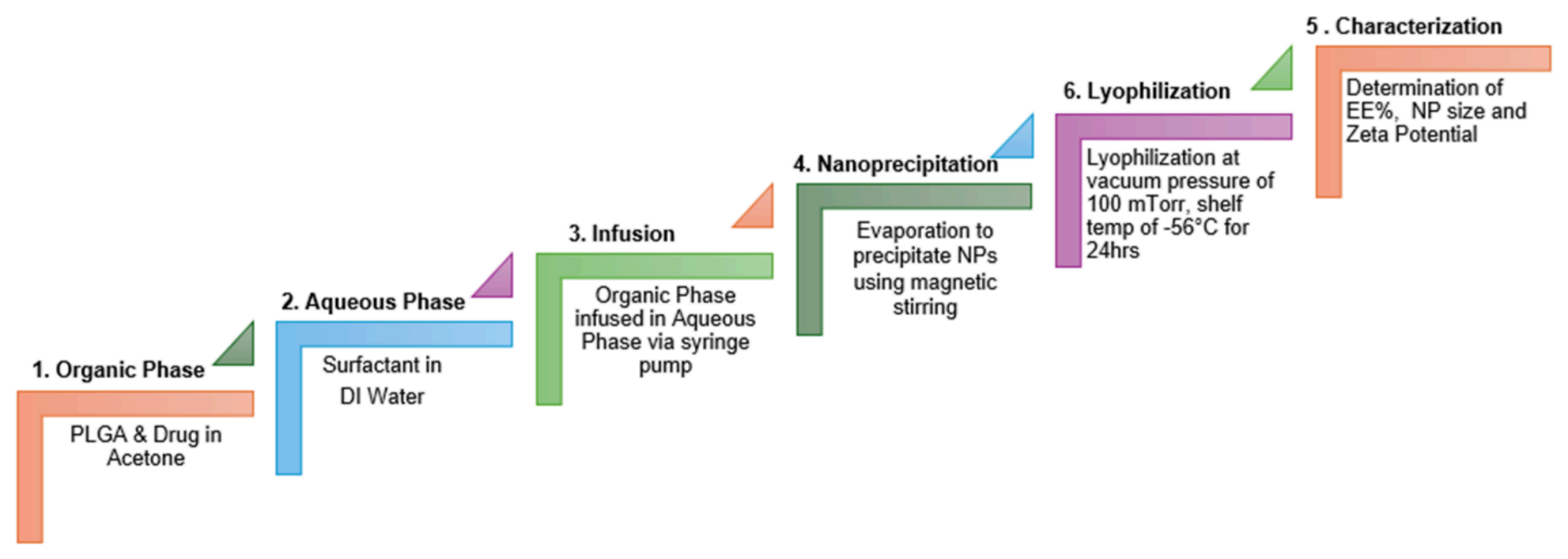
Schematic illustration of key steps in the nanoprecipitation process.

Practical application of DOE in pharmaceutical formulation development was demonstrated using a poly(lactic-co-glycolic acid) nanoparticles (PLGA NPs) case study. This case study illustrates the optimization of PLGA NPs synthesized via the nanoprecipitation technique (shown in Figure 11) to achieve precise control over formulation characteristics. The poorly water-soluble AC1LPSZG, a novel synthetic mTORC1/2 inhibitor with promise for treating non-small cell lung cancer (NSCLC), was used as the model drug [176].

**Figure 12:**
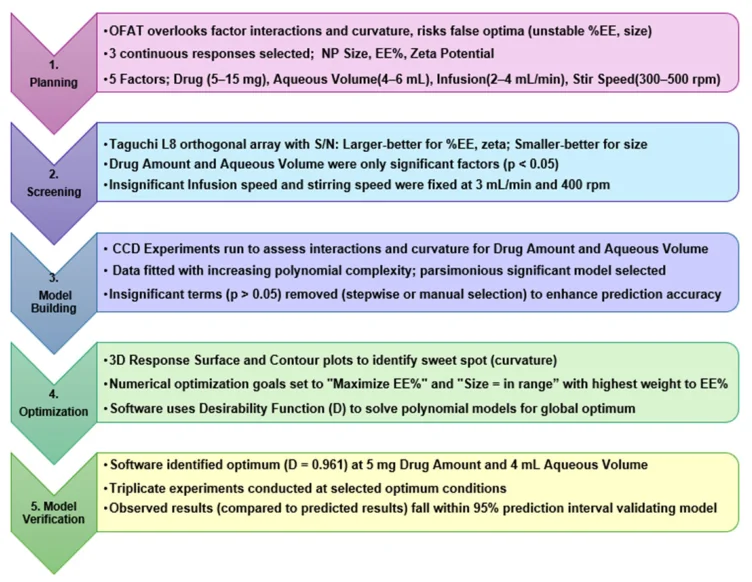
Five-step DOE workflow applied to PLGA NP formulation, illustrating systematic progression from factor screening (Taguchi design) to model development (central composite design), numerical optimization, and experimental validation.

The five-stage DOE workflow for NP formulation optimization advances from problem definition and Taguchi screening to CCD optimization using quadratic models (Figure 12).

### 5.1. Planning

The planning step in DOE involves identifying the problem, defining clear objectives, selecting measurable responses, and choosing relevant factors, levels, and potential interactions to be evaluated.

#### 5.1.1. Problem Statement

Conventional OFAT methods cannot account for the interdependent factors and their interactions. This leads to a “false optimum,” where researchers have to settle for suboptimal quality (i.e., low EE% and inconsistent particle sizes).

#### 5.1.2. Specific Objective

The objective of this case study is to employ a multistage DOE approach—moving from a Taguchi L_8_ screening to a central composite design (CCD)—to identify the precise “sweet spot” where %EE is maximized and particle size is controlled within a nano range. By applying advanced diagnostics like Box–Cox power transformations in Design Expert^®^ software, the study aims to build a validated mathematical model capable of accurately predicting NP formulation characteristics.

#### 5.1.3. Responses and Measurement Systems

Three response characteristics were measured—entrapment efficiency (EE%), NP size, and zeta potential. The Malvern Zetasizer Nano-ZS (Model: ZEN 3600) from Malvern Instrument Ltd., Worcestershire, UK, was utilized for particle size and zeta potential measurements. Entrapment efficiency (EE) was assessed using the previously published method on the Waters Acquity UPLC system [177].

#### 5.1.4. Factors and Factor Levels

The factors and factor levels for preparation of PLGA NPs using the nanoprecipitation method were identified based on published literature [80,178,179]. Four factors were examined, and factor levels were set as follows: A: drug amount (5–15 mg), B: aqueous volume (4–6 mL), C: infusion speed (2–4 mL/min), and D: stirring speed (300–500 RPM).

### 5.2. Screening

The Taguchi L_8_ orthogonal array was used to narrow down important factors before high-resolution optimization. Unlike conventional DOE, which focuses on average (mean) performance, the Taguchi method focuses on how variations (fluctuations/noise) affect a process [180]. Using orthogonal arrays, it efficiently identifies factor levels at which the NP formulation is insensitive to noise, making them robust against variations. Signal-to-noise (S/N) ratios measure factor robustness, while the delta value (max–min) ranks factor effects. The Taguchi method also quantifies each factor’s contribution, ensuring high-quality performance with significantly fewer experimental runs than full factorial designs.

#### Taguchi L_8_ Orthogonal Array

A Taguchi L_8_ orthogonal array (Table 5) was initially employed to screen four factors at two levels using only eight runs, compared to 16 runs required for a full factorial design. The Taguchi L_8_ array is a balanced orthogonal design, as each factor has two levels and each level appears exactly four times (balanced). For any pair of factors, all level combinations occur equally (e.g., for the factor pair A vs. B—(5,4), (5,6), (15,4), (15,6)—each level combination appears two times). This is a Resolution III screening design ensuring clear main effects while main effects are confounded with interactions.

Each row of the L_8_ screening matrix represents one experimental run, executed in randomized order. Taguchi results are analyzed using ANOVA (*p*-value and % contribution), and S/N ratios to determine factor importance (S/N ratios for individual responses were calculated manually using Excel (version 2608) and Minitab (version 22) softwares). Influential factors with larger effects or higher variance contribution (often >5%) are kept for further optimization, while those <5% of total SS are pooled into error [47].

ANOVA (Table 6) identifies drug amount (A) as a highly significant factor (*p* < 0.01) dominating with >86% contribution across all responses. Aqueous volume (B) critically controls solvent diffusion in the nanoprecipitation process, contributing 13% to NP size, though it does not significantly affect EE% (*p* = 0.1915) or zeta potential (*p* = 0.1245). Infusion (C) and stirring speeds (D) seem to be less important factors contributing <5% to variance; any significance might be attributed to two-factor interactions (confound in this Resolution III design). It suggests that tested infusion rates (2–4 mL/min) enabled rapid nucleation, while 400–500 RPM ensured adequate mixing, justifying their exclusion from further optimization.

NP formulation robustness was assessed using S/N ratio criteria “Smaller-the-Better” for NP size and “Larger-the-Better” for EE% and zeta potential (magnitude). Table 7 provides the statistical hierarchy (ranks) for all parameters. Drug amount (Rank 1) dominates with the largest delta values for both mean and S/N ratio (EE%: Mean = 22, S/N = 7.37), serving as the primary driver for NP formulation performance and consistency. Infusion and stirring showed minimal deltas (lowest ranks) in general, confirming process robustness against variations in these parameters.

This screening phase provided a clear distinction between influential formulation variables (Factor A and B) and robust mechanical parameters (Factor C and D). Consistent ranking and high contributions identified drug amount and aqueous volume as key NP quality drivers, justifying a central composite design (CCD) to map their complex interactions and quadratic effects. Insignificant factors, infusion (C) and stirring speeds (D), were fixed at their mean levels (3 mL/min and 400 RPM), simplifying the design while ensuring robust and scalable NP formulations.

### 5.3. Optimization

Post-screening phase, a central composite design (CCD) was established to generate a high-resolution map of the significant factors—drug amount and aqueous volume. Since a Box–Behnken design (BBD) requires three factors, CCD was chosen to quantify curvature and optimize %EE within this two-factor study.

#### 5.3.1. Central Composite Design (CCD)

Two independent variables (factors)—A: drug amount in the organic phase (mg) and B: aqueous phase volume (mL)—were explored across five levels (−1, 0, +1, −α, and +α), as shown in Table 8. The CCD design layout and three measured responses—EE%, NP size, and zeta potential—are recorded in Table 9. For k number of factors and n center points, the total number of experiments in CCD is given by Equation (7):

N* = 2ᵏ + 2*k* + *n* (7)

where 2*ᵏ* represents the factorial points (±1, ±1) used to estimate linear and interaction effects (for *k* = 2, 2^2^ = 4 factorial points); 2*k* represents the axial points [(±α, 0) and (0, ±α)] added to estimate quadratic effects (for *k* = 2, 2 × 2 = 4 axial points); and n denotes the number of center points (0, 0) included to estimate pure experimental error.

In CCD, the alpha (α) value represents the distance of the “axial” or “star” points from the center of the design space. Axial distance α = 1.41 was used in this case study to achieve a rotatable CCD (ensuring constant prediction variance at all points equidistant from the center). A total of four center points, coded as (0, 0), are added in this case study. In this specific twelve-run CCD, the factorial core (±1 levels) is orthogonal, so main effects and interactions are well separated. However, quadratic effects are not orthogonal to each other, meaning they are partially confounded with each other and the intercept. The experimental runs were randomized to identify time-related variations (temperature and humidity) and to distribute the effects of noise throughout all experiments.

**Table 8:** Correspondence between coded factor levels and actual experimental values within the central composite design (CCD) framework.

	Coded Factor Levels				
Factors	Low (−1)	Mean (0)	High (+1)	Min (−α)	Max (+α)
A: Drug Amount in Organic Phase (mg)	5	10	15	2.93	17.07
B: Aqueous Phase Volume (mL)	4	5	6	3.59	6.41

**Table 9:** Central composite design (CCD) layout for optimization of AC1LPSZG-loaded PLGA NPs and measured responses. Points: factorial (−1, +1), center (0), axial (−α, +α).

Run	Coded Factors	Space Type	EE *(*%)	NP Size (nm)	Zeta Potential (mV)
1	(0, 0)	Center	19	142	−17
2	(0, 0)	Center	20	140	−14
3	(+α, 0)	Axial	15	135	−16
4	(−α, 0)	Axial	59	152	−18
5	(+1, +1)	Factorial	21	143	−15
6	(0, −α)	Axial	16	139	−13
7	(0, 0)	Center	19	159	−19
8	(0, 0)	Center	17	145	−21
9	(−1, −1)	Factorial	38	134	−20
10	(−1, +1)	Factorial	22	178	−21
11	(0, +α)	Axial	30	156	−17
12	(+1, −1)	Factorial	13	146	−17

Coefficient of determination (R^2^) is a measure of total variability explained by the chosen model. Adjusted R^2^ assesses model fit accounting for insignificant predictors, while predicted R^2^ evaluates predictive reliability for new data. ⁽^1^⁾ Adequate Precision is NA for mean models because constant predictions yield the signal range zero and the ratio mathematically incalculable.

PRESS, predicted residual error sum of squares; BIC, Bayesian information criteria; AIC, Akaike’s information criteria; −2LL, −2 log likelihood.

Cor Total, corrected total (indicates sum of squares (SS) corrected for the mean, not zero baseline).

* Assuming normal distribution, approximate 95% PI = predicted mean ± 1.96 × SD.

#### 5.3.2. Model Selection

Design-Expert^®^ version 13 automates RSM model selection by sequentially fitting polynomial models (Mean → Linear → 2FI → Quadratic) using least-squares regression for responses (EE%, NP size, and zeta potential). Models are validated via ANOVA, lack of fit, and AIC, selecting the highest-order significant model that minimizes AIC (penalizing complexity) while maintaining hierarchy [49]. Model adequacy is confirmed by a high R^2^ (close to 1), with a close gap between adjusted and predicted R^2^ (<0.2 difference), CV < 15%, adequate precision (signal/noise > 4), and non-significant lack of fit (*p* > 0.05).

#### 5.3.3. EE (%) of PLGA NPs

EE% ranged from 13% to 59% (Table 9). Sequential model selection identified the two-factor interaction (2FI) model as superior to mean and linear options with a high R^2^ of 0.973 (i.e., 97.3% variability is explained by the 2FI model) (Table 10). Lowest PRESS and AIC values confirmed predictive strength of selected model (Table 11). ANOVA (Table 12) confirmed a highly significant model (*p* < 0.0001), with all terms (A, B, and AB) significant (*p* < 0.0001), indicating a negligible probability (0.01%) of model significance arising from noise. Calculated model F-value of 94.29 far exceeded the critical threshold (4.07), demonstrating overall model significance. A non-significant lack of fit (*p* **=** 0.9002, F = 0.2759) proved the model accurately fits the data against pure error. Model reliability was excellent with an adjusted R^2^ of 0.962 ≈ predicted R^2^ of 0.947 (gap < 0.2), an adequate precision of 30.3 (>4), and a CV of 14.6% (<15%).

**Figure 13:**
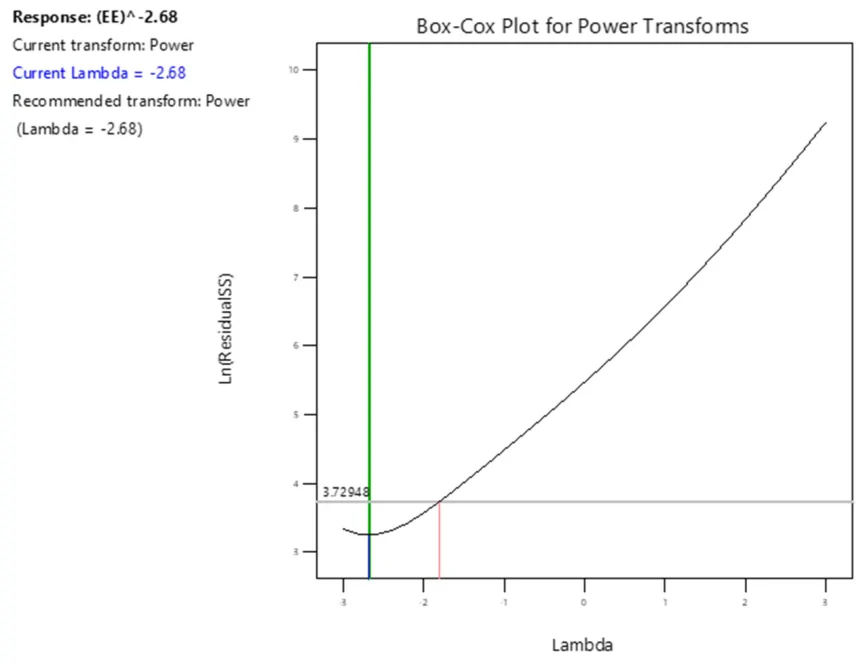
Box–Cox plot for power transformation of EE% data. The plot illustrates the recommended lambda (λ) value of −2.68, identified at the minimum point of the ln (residual SS) curve, to ensure the statistical validity of the response surface model.

To satisfy the assumption of normality, a Box–Cox power transformation with lambda (λ) = −2.68 was applied to EE% data (Figure 13). This yielded the following coded stepwise regression model (Equation (8)) based on CCD response data in Table 9:

(8) $${\left(EE\right)}^{-2.68}=0.00039+0.00025A-0.00016B-0.00024AB$$

**Figure 14:**
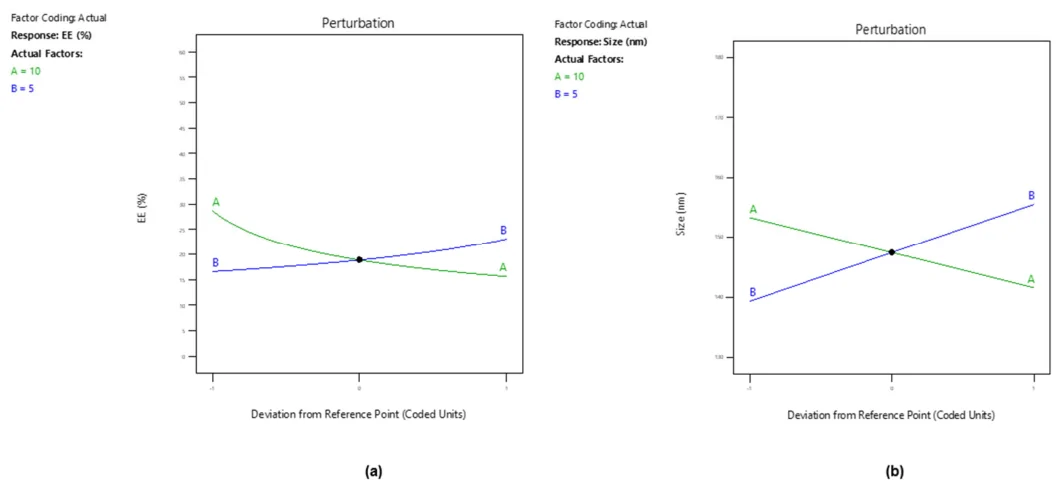
Perturbation plots depict the sensitivity of (**a**) EE% and (**b**) NP size to individual factors. The plots illustrate response changes as drug amount (Factor A, green line) or aqueous volume (Factor B, blue line) deviates from the center point across the design range (−1 to +1), holding the other factor constant (at mid-level).

ANOVA sum of squares (SS) (Table 12) and coefficient magnitudes in Equation (8) identified drug amount (A) as the main driver, followed by interaction AB, while aqueous volume (B) had the least effect. Due to the inverse transformation (EE)^−2.68^, coefficient signs reflect opposite actual effects on EE%. Increasing drug amount (A) reduces EE%, whereas increasing aqueous volume (B) improves it. The significant AB interaction shows combined effects differ from individual factors, with higher aqueous volume mitigating the negative impact of high drug amount. The perturbation plot (Figure 14a) highlights similar trends. Increasing drug amount (A) sharply reduces EE% (from ~29% to ~17%), while aqueous volume (B) moderately improves it (from ~17% to ~24%) as factors shift from −1 to +1 levels. The AB interaction further modifies these slopes, showing that the influence of one factor shifts depending on the level of the other.

**Figure 15:**
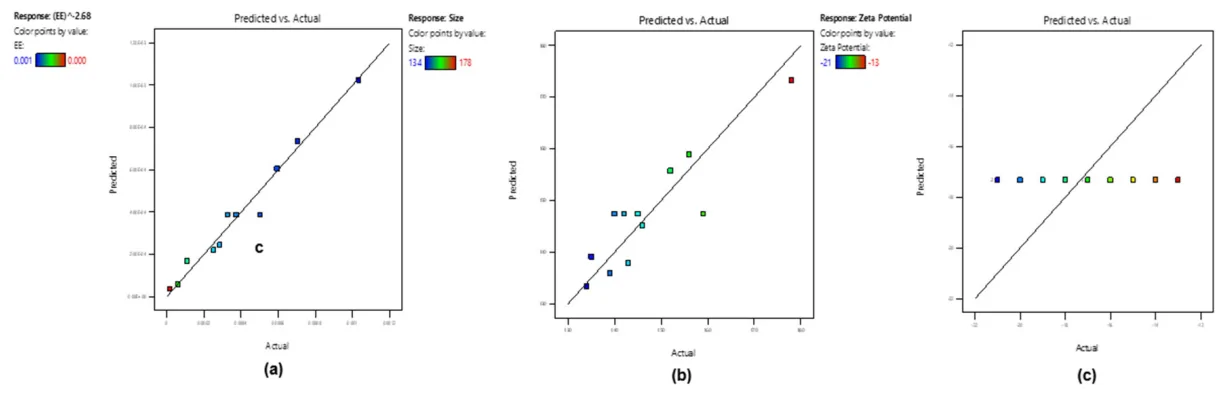
(**a**) Plot of predicted versus actual response. (**a**) % EE, (**b**) size, and (**c**) zeta potential. The tight clustering of data points around the diagonal line indicates a strong correlation between the model predictions and the experimental results, confirming the model’s accuracy (goodness of fit). The horizontal alignment of the zeta potential data points indicates that the model failed to detect any factor-induced variability, implying the response was unaffected by the tested variable ranges.

**Figure 16:**
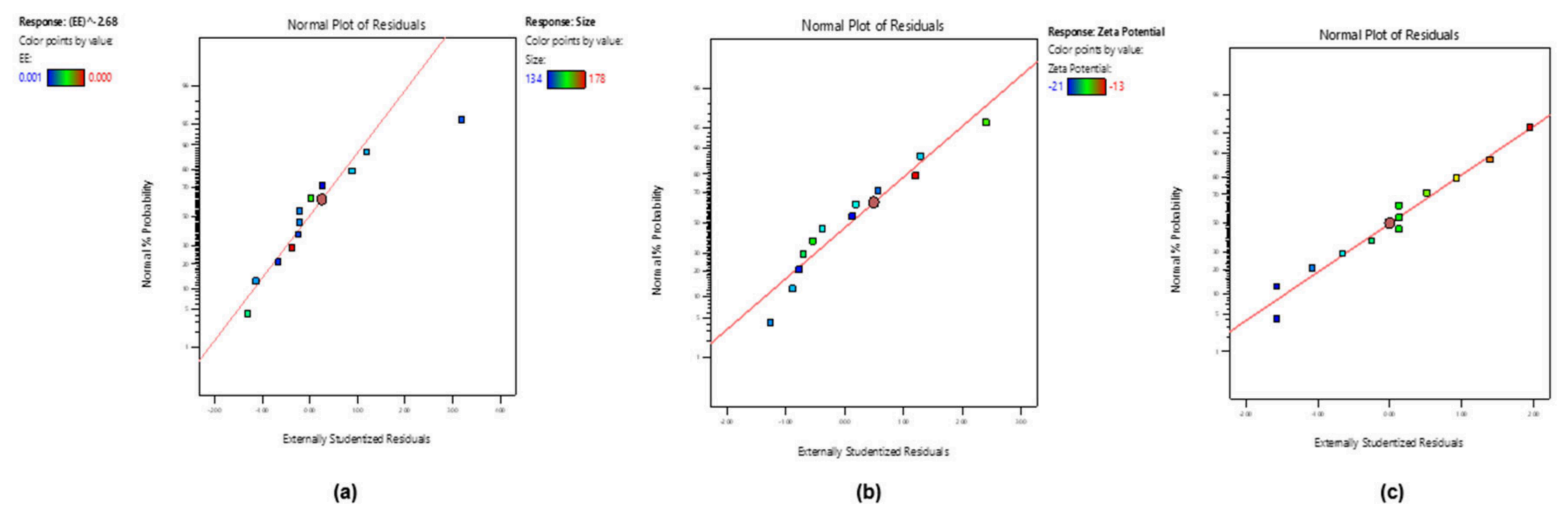
Normal probability plot of the internally studentized residuals. (**a**) % EE, (**b**) size, and (**c**) zeta potential. The data points follow a linear trend line, validating the assumption that the error terms (residuals) are normally distributed.

**Figure 17:**
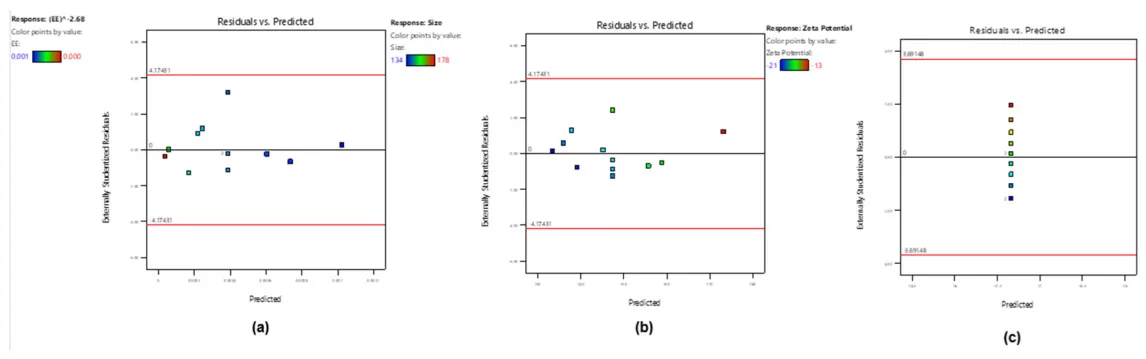
Residuals vs. predicted plot. (**a**) % EE, (**b**) size, and (**c**) zeta potential. The containment of all data points within the red outlier detection limits confirms homoscedasticity of residuals and the absence of influential outliers. The vertical alignment of all zeta potential data points indicates that the model provides a constant prediction regardless of the observed variation.

**Figure 18:**
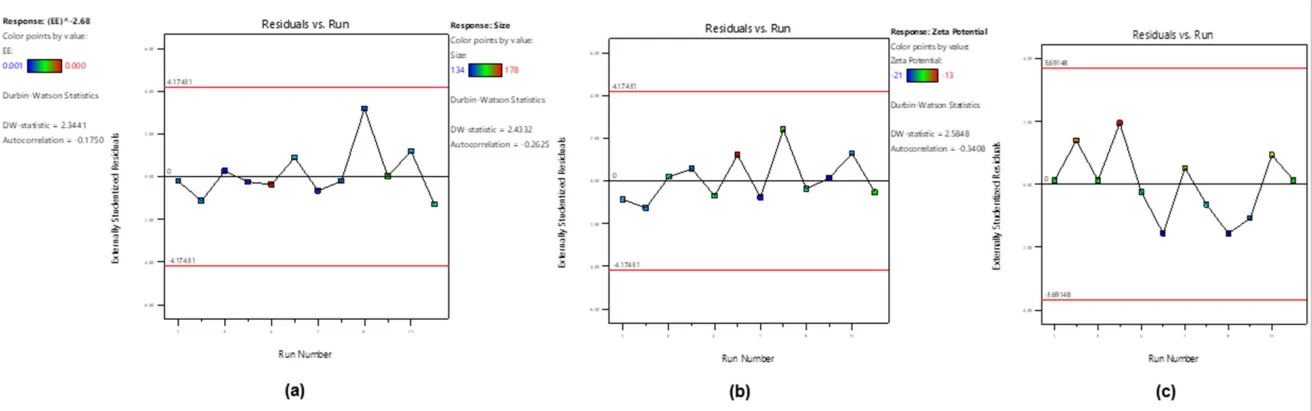
(**a**) Residuals vs. run plot. (**a**) % EE, (**b**) size, and (**c**) zeta potential. The random distribution of residuals within the red control boundaries indicates that the model is free from time-related bias or lurking variables.

Model diagnostics confirmed the 2FI model’s adequacy by satisfying all ANOVA assumptions. The predicted vs. actual plot (Figure 15a) shows high correlation, with data points tightly clustered along the line of identity. Three key plots of residuals (i.e., quantitative differences between observed and predicted values) further validated the model’s reliability. The normal probability plot (Figure 16a) displays a linear pattern, confirming normally distributed error terms, ensuring valid *p*-values and F-statistics derived from the ANOVA test. The residuals vs. predicted plot (Figure 17a) was evaluated after Box–Cox power transformation (lambda = −2.68) to confirm that the issue of non-constant variance (heteroscedasticity) was effectively stabilized. It confirmed a random scatter of residuals around the zero-reference line (no funneling or funnel or megaphone structure), confirming constant variance or homoscedasticity. All data points fall within the ±3 standard deviation control limits (upper and lower red horizontal lines), confirming no outliers or leverage points significantly skewing the model. This ensures the model maintains equal predictive consistency across all response range without bias. Residuals vs. run plot (Figure 18a) shows no systematic time-related trends (steady climb or decline), and all residual values remained within the ±3 standard deviation control limits. It supports the assumption of independent errors without any time effects or “lurking variables.” During the experiment, key environmental conditions such as ambient temperature and humidity were monitored, and no evidence of their influence was observed, supporting the conclusion that the model is free from these significant lurking variables.

**Figure 19:**
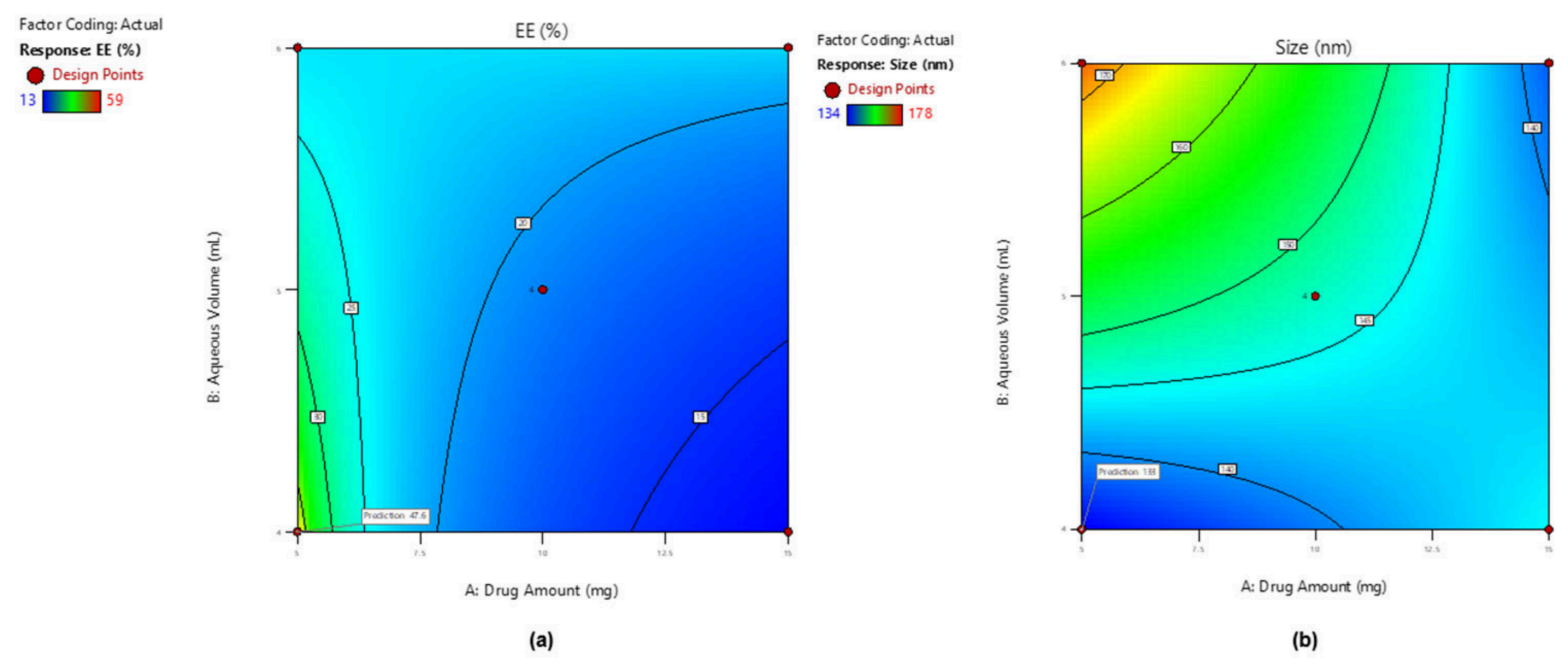
2D contour plots illustrate the interactive effects of drug amount (A) and aqueous volume (B) on (**a**) EE% and (**b**) particle Size. The colored gradients represent the response magnitude, transitioning from lower (blue) to higher (green/red) values.

**Figure 20:**
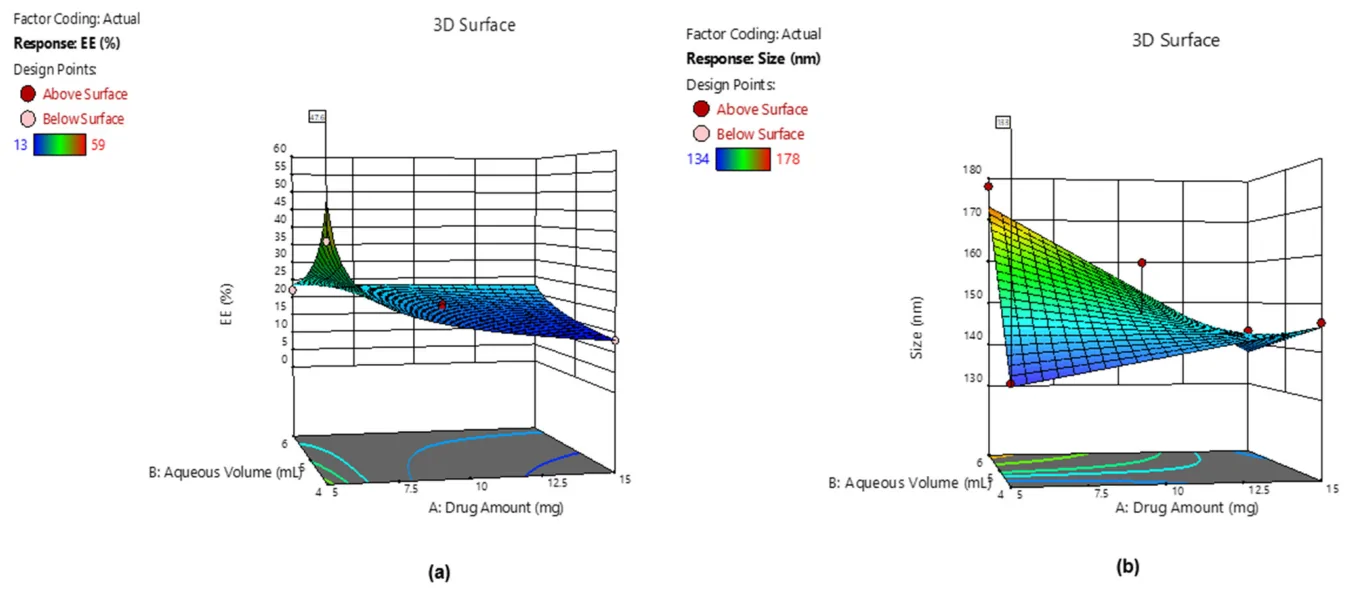
3D response surface plots depicting the influence of drug amount (A) and aqueous phase volume (B) on NP characteristics: (**a**) EE% and (**b**) particle size. The continuous surface curvature highlights the significant interaction effects between variables, facilitating the identification of the optimal design space.

Contour plot and 3D response surface plot graphically illustrate the regression model (represented by Equation (8)). The contour plot (Figure 19a) maps two factors (A: drug amount and B: aqueous volume), with equal EE% values connected as contour lines; closer lines indicate steep changes. The central dots denote four center point replicates. Colored bands illustrate response ranges: blue bands indicate lower EE%, and green bands represent higher values. The highest EE% is projected at the bottom-left corner, where both factors are at their lower levels. The 3D response surface plot (Figure 20a) provides a continuous representation of this relationship. By plotting factors A and B on the horizontal axes (X1 and X2) and EE% on the vertical axis (Y), the surface visually captures the interaction effects, confirming optimal low-level conditions.

#### 5.3.4. Size of PLGA NPs

All synthesized NPs remained within the nanometric range (134–178 nm, Table 9). The 2FI model provided the best fit, showing strong statistical performance with an R^2^ = 0.803 (Table 10), explaining 80.3% of NP size variability. The close values of adjusted R^2^ (0.728) and predicted R^2^ (0.600) confirm good predictive ability (difference < 0.2). Lowest AIC and PRESS values (Table 11) further supported balanced, non-overfitted model selection.

ANOVA (Table 13) confirms the 2FI model is highly significant for NP size. The overall model F-value of 10.84 (critical F-value of 4.07) with a *p*-value of 0.0034 (*p* < 0.05) indicates a statistically significant model, with only a 0.34% probability that the observed significance is due to random noise.

While drug amount (A) is significant (*p* = 0.0311), aqueous volume (B) and the AB interaction show much stronger influence (*p* < 0.01). The lack of fit was insignificant (F **=** 0.2651, *p* = 0.9065), proving the model accurately captures factor relationships without structural flaws. Strong model metrics—adequate precision of 10.6 (>4) and CV% of 4.4 (<15%)—indicate high precision and reliability, validating the 2FI model’s robustness (Table 10).

Equation (9) describes the 2FI model for NP size, with an intercept of 147.32 nm representing the mean response. The aqueous volume (B) has a dominant influence on NP size, with a larger coefficient magnitude (+8.19) than drug amount (A) (−5.98). Perturbation plot (Figure 14b) shows that increasing drug amount reduces NP size from 154 nm to 140 nm, while increasing aqueous volume increases size from 139 nm to 158 nm as factors change from −1 to +1 levels. The steeper B slope confirms a stronger impact of aqueous volume. The AB interaction is the most critical term (*p* = 0.0070), accounting for ~32% of the total variability in NP size (SS_AB_/SS_Total_) and ~40% of the model-explained variability (SS_AB_/SS_Model_). Its large negative coefficient (−11.65) indicates that combining high A and high B reduces size more than predicted by individual effects alone.

(9) Size=147.32−5.98A+8.19B−11.65AB

Diagnostic plots confirm the 2FI model’s robustness. Predicted vs. actual plot (Figure 15b) aligns tightly with the identity line, showing high accuracy. Normal probability plot (Figure 16b) shows residuals following the normality assumption. Residuals vs. predicted plot (Figure 17b) displayed random scatter of residuals around the zero-reference line, validating homoscedasticity. Residuals vs. run plot (Figure 18b) lacks trends, ruling out time/sequence effects, ensuring an unbiased model.

Contour plot (Figure 19b) reveals that minimal NP sizes (blue regions) occur at two opposite factor extremes (both A and B low or both A and B high), forming a “saddle” shape due to the strong AB interaction. The 3D surface plot **(**Figure 20b) clarifies this saddle effect, where curvature reverses across axes, yielding optima at opposite corners (bottom-left and top-right) rather than a central minimum. This “saddle” geometry causes factor sensitivity to flip, proving that a one-factor-at-a-time (OFAT) approach would fail to find the true optimal settings for minimizing NP size.

The saddle-shaped response surface for NP size reveals a highly interdependent relationship between drug amount (A) and aqueous volume (B). The strong negative AB interaction coefficient (−11.65) dominates precipitation kinetics over individual main effects. Consequently, minimal NP sizes emerge at two opposite corners of the design space—when both factors are simultaneously low (−1, −1) or simultaneously high (+1, +1).

At the low–low condition, the system operates via kinetically restrained nanoprecipitation. Because the drug amount (A) is low, fewer molecules are available for aggregation. This state favors homogeneous nucleation with limited growth. Although the aqueous volume is small, insufficient solute exists to sustain prolonged growth, resulting in limited particle enlargement and smaller NPs. Nuclei form rapidly, and because the solute reservoir is quickly exhausted, the kinetics freeze before significant collision-mediated aggregation, or Ostwald ripening, can occur.

Conversely, the high–high extreme triggers diffusion-driven rapid nucleation. A high drug amount generates intense supersaturation and increases the nucleation rate exponentially. This creates a massive burst of tiny embryonic nuclei. However, simultaneously large aqueous volume acts as an anti-solvent sink that accelerates solvent diffusion and rapidly dilutes the system. This rapid dilution effectively suppresses particle growth and prevents aggregation. It isolates newly formed nuclei (by increasing the physical distance between them), lowers collision frequency, and produces minimal nanoscale particles.

#### 5.3.5. Zeta Potential of PLGA NPs

ANOVA (Table 14) confirms the mean model as the best fit for zeta potential, indicating that neither drug amount (A) nor aqueous volume (B) has significant effect on surface charge.

All variation (Cor Total SS: 75.70) stems from random error—“Pure Error” or “Lack of Fit”—rather than systematic factor effects (model SS: 0.00). Non-significant lack of fit (*p* **=** 0.6942) confirms noise-driven variation and stable-surface properties for mean model. The stable, uniform zeta potential suggests surface charge might be governed by constant (unexplored) variables like polymer or surfactant type, regardless of factor levels. As no numeric factors were significant, the response surface is a horizontal flat plane with zero slope, omitting perturbation/contour/surface plots. Regression metrics like R^2^ and adequate precision are not applicable (Table 10), as there is no correlation to measure between the factors and the response.

#### 5.3.6. Mathematical (Numerical) Optimization and Model Verification

Mathematical (numerical) optimization was applied to identify optimal levels of drug amount (A) and aqueous volume (B) for the NP formulation, followed by a verification step to confirm the model’s reliability. In Design-Expert^®^, response priorities were adjusted using the importance settings (from 1+ to 5+), with %EE set to “maximize” at the highest priority of 5+ (+++++) to ensure high therapeutic payload, while particle size and zeta potential were kept “in range.” Crucially, particle size and zeta potential were not ignored; they were constrained to remain “in range.” This restriction was applied because all experimental formulations fell within the desired nanoscale range required for effective tumor targeting, and the zeta potential consistently followed a stable mean model, indicating excellent colloidal stability in this particular example.

**Figure 21:**
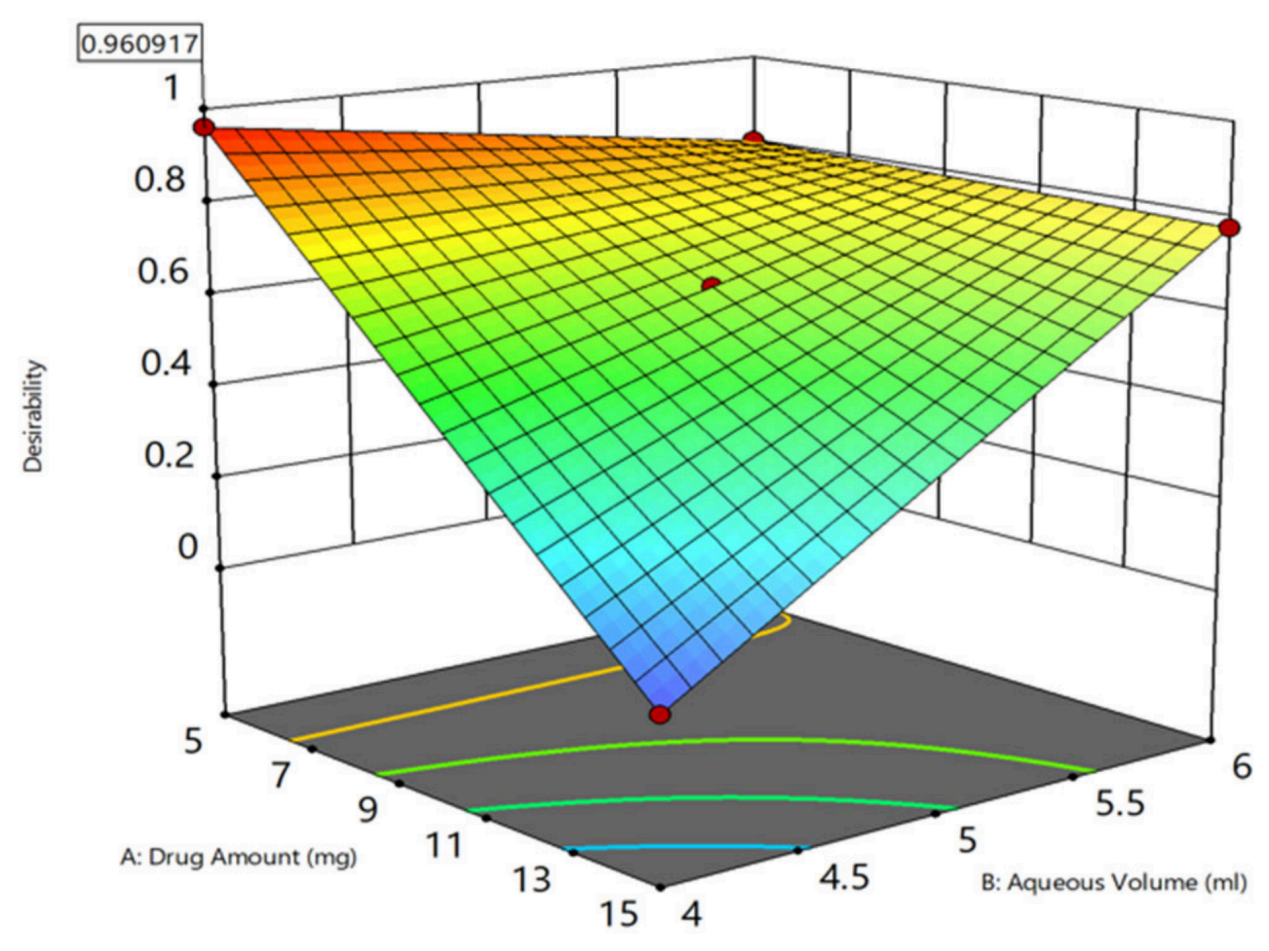
Desirability plot to identify optimized NP formulation region—predicted optimal factor A and B coordinates along with corresponding global desirability score (combined for all responses). The color gradient represents desirability values from lowest to highest: blue < green < yellow < red, where blue indicates lower desirability and red represents higher values.

The software generated multiple optimization solutions ranked by desirability (Table 15), with the top solution (desirability = 0.961; Figure 21) corresponding to 5 mg of drug and 4 mL of aqueous volume. This score indicates that the suggested coordinates almost perfectly satisfy the combined requirements of NP formulation.

To confirm the model’s predictive accuracy, three independent replicates were synthesized at these optimized coordinates (row 1 in Table 15). The experimental results were then compared against the 95% prediction intervals (PI), thereby assessing the adequacy of the optimization model (Table 16). The confirmatory NPs showed mean values of EE 41.2%, NP size 124 nm, and zeta potential −15 mV, which were in good agreement with the corresponding predicted values (EE 47.6%, NP size 133 nm, and zeta −17 mV), supporting the reliability of the numerical optimization and model verification approach. Since all observed responses fall within their respective 95% PIs, the model is successfully validated and confirmed adequate for predicting new data.

#### 5.3.7. Process Sensitivity Analysis and Scale-Up Considerations

Based on the numerical optimization results in Table 15, the formulation showed high sensitivity to changes in aqueous volume (B), a critical process parameter (CPP), while the drug amount (A) remains stable at its low boundary of 5.0 mg across the top six predictive models. A slight increase in aqueous volume (B) reduced encapsulation efficiency (%EE) and desirability D, indicating the need for strict process control during scale-up. We can look at the transition from the optimized Solution 1 (Selected) to Solution 2.

**Baseline (Solution 1):** Drug amount = 5.0 mg, aqueous volume = 4.0 mL. This yields an encapsulation efficiency (EE) of 47.6%, an NP size of 133 nm, and an overall desirability of 0.961.

**Deviation Analysis (Solution 2):** An increase in aqueous volume from 4.0 mL to 4.7 mL represents a shift in the process space. Analyzing the localized gradient between Solution 1 and Solution 2 reveals:

- **Desirability Penalty:** A minor increase in aqueous volume causes the overall geometric desirability to drop significantly from **0.961 to 0.903.**
- Impact on Quality Attributes (CQAs): This shift causes a sharp 34.4% relative decrease in %EE (dropping from 47.6% to 31.2%) and a simultaneous 11.3% increase in NP size (enlarging from 133 nm to 148 nm). Zeta potential remains entirely unaffected at −17.2 mV.

This sensitivity analysis reveals that the optimized formulation is highly sensitive to aqueous volume. Small deviations can significantly reduce EE%, increase NP size, and lower overall desirability. Therefore, strict control of aqueous volume is essential during industrial scale-up, justifying the selection of Solution 1 as the optimal condition.

## 6. Future Roadmap: AI-Augmented DOE for Nanomedicine Development

Artificial intelligence (AI) is a computational system designed to mimic human intelligence, while machine learning (ML) refers to algorithms that learn from data. Integrating AI/ML with DOE is transforming nano-pharmaceutical development by enabling predictive modeling, formulation optimization, and biological performance assessment. Deep learning further enhances complex data analysis, prediction accuracy, and reproducibility [6]. AI/ML algorithms can analyze multidimensional formulation datasets to identify complex relationships among critical material attributes (CMAs), critical process parameters (CPPs), and critical quality attributes (CQAs). DOE provides structured experimental designs to generate high-quality data for AI/ML model training, validation, and optimization. This integration defines a multidimensional design space, reducing variability, improving scale-up, and supporting regulatory compliance for complex nanomedicines. This synergy dramatically reduces experimental effort, accelerates factor screening, and predicts complex formulation behavior, enabling robust optimization of NP formulations [181]. Recent research demonstrated that ML improves DOE by capturing complex nonlinear formulation–response relationships, with k-nearest neighbors (KNN), a data-driven ML algorithm, showing strong predictive performance for ZnO nanoparticle optimization. By implementing advanced ML algorithms such as random forest, KNN, and extreme gradient boosting (XGBoost), this data-driven computational approach excels at capturing localized data structures and intricate, multi-variable interactions [182].

Artificial neural networks (ANNs) simulate brain-like learning using hundreds of artificial neurons (single units) organized into interconnected computational layers (input, hidden, and output layers). Data enters the network through the input layer, which receives the independent variables (input factors), while the final output layer predicts the dependent variables (responses). One or more hidden layers lie between the input and output layers [183]. Each neuron processes complex nonlinear relationships through weighted connections and activation functions. During training with DOE-generated datasets, learning algorithms iteratively adjust connection weights to minimize prediction error. Once fully trained and validated, ANNs accurately predict novel formulation responses, effectively supporting robust DOE-based optimization [184]. ANNs learn complex relationships directly from experimental data, moving beyond rigid statistical models to flexible, data-driven computation. Combined with DOE, they improve prediction accuracy, optimization, and decision-making [178].

## 7. Conclusions

DOE provides a robust, structured framework for optimizing NP formulations, allowing researchers to achieve superior outcomes with minimal material and time investments, while powerful, successful DOE implementation requires careful planning, appropriate design selection, and rigorous model validation to ensure reliable and reproducible outcomes. This review highlighted core DOE principles, practical implementation strategies, and common pitfalls, emphasizing their relevance to QbD-driven pharmaceutical formulation development. The PLGA NP case study illustrated how a multistage DOE framework—from screening to response surface optimization—can identify critical variables, generate predictive models, and deliver experimentally validated outcomes. Consequently, this framework empowers researchers to efficiently navigate complex design spaces, ensuring the development of stable, reproducible, and scalable resource-efficient NP formulations. Overall, this work serves as a practical reference for pharmaceutical scientists, particularly early-career researchers, seeking to translate DOE outputs into robust, data-driven decisions that accelerate NP formulation development. Ultimately, broader DOE adoption can drive faster innovation and efficiency in the pharmaceutical industry and research. Beyond this, AI-augmented DOE is transforming nanomedicine development through predictive, data-driven formulation optimization with fewer experiments. Future integration of AI and ANN will accelerate NP design, improve robustness, and support scalable, regulatory-ready nanotherapeutic development.

## Figures and Tables

**Table 1:** Factor kevels required for different order polynomials.

Polynomial Order	Required Levels	Curve Shape
First order or Linear	2	Straight line (shows a constant increase or decrease)
Second order or Quadratic	3	Hill/valley (standard for finding an optimum)
Third order or Cubic	4	S-curve (can show a peak followed by a valley)
Fourth order or Quartic	5	W (or M)-shape (used for complex, multimodal systems)

**Table 2:** Overview of common DOE: key features, applications, and limitations.

Design	Factor Type	Key Features	Runs (Example)	Resolution/Aliasing	Applications	Limitations
Full Factorial [99,100,101,102,103,104,105]	Both, categorical and continuous	Tests all combinations of *k* factors (continuous, numeric, or categorical) at two or three levels, with optional partial or full replications to estimate pure error and perform lack-of-fit tests	Two- and three-level full factorial designs require $2^{k}$ and $3^{k}$ runs, respectively (i.e., $2^{4}=16$ runs for four factors at two levels each)	Infinite (no confounding)	Estimate all main effects and all possible interactions; simple interpretation; robust design for small factor numbers	Large experiments become impractical with many factors (use screening designs instead)
Fractional Factorial [49,60,99,106,107,108,109,110]	Both, categorical and continuous	Tests a subset of full-factorial combinations; runs some fraction (1/2, 1/4, 1/8, etc.) of full factorial design; trades resolution for efficiency	Runs = 2^k−*p*^ for two-level; runs = 3^k−*p*^ for three-level fractional factorial designs, where k is factor number and *p* represents fraction of full factorial design 2^5−1^ is half fraction of 2^5^ to test five factors in 16 runs 2^6−2^ is quarter fraction of 2^6^ to test six factors in 16 runs	III–V 2^3−1^ is Resolution III (three factors in four runs) 2^4−1^ is Resolution IV (four factors in eight runs) 2^5−1^ is Resolution V (five factors in 16 runs)	Screen many factors (when higher interactions are negligible) and identify significant factors for further investigation	Cannot estimate all interactions; multilevel fractional factorial designs are cumbersome (use Latin squares or Taguchi method instead)
Box Wilson Central Composite Design (CCD) [82,83,84,85,111,112,113,114]	Continuous, categorical (only via blocking and replicating design)	Robust to missing data; facilitates sequential experimentation; three variants—CCC (circumscribed): Original CCD, five levels, rotatable, precise quadratic estimates; CCI (inscribed): scaled-down CCC, five levels, rotatable, axial points contracted (±1); CCF (face-centered): Simpler, three levels, not rotatable, limited quadratic estimates	For k factors and n center points, total experiments N* = 2ᵏ + 2k + n (2ᵏ factorial points for linear/two-factor effects, 2k axial points for quadratic terms, and n center points for pure experimental error); design is repeated for categorical factor levels	V (2FI clear)	Captures curvature, interactions, and quadratic effects—five levels in CCDs can test up to a fourth-order model (avoiding full three-level factorial design); robust for optimization and modeling; good prediction	Factorials runs (2ᵏ) grow exponentially, driving up costs; extreme axial points may lie outside safe range; running five-factor levels (low, high, center, and two axial points) can often be impractical; quadratic terms may lack orthogonality
Box–Behnken Design (BBD) [86,87,89,115,116,117]	Continuous	Independent quadratic design with no embedded factorial core and avoids simultaneous extreme points (unlike CCDs); needs ≥3 factors to evaluate quadratic effects by placing points at the midpoints of edges of a 3D cube	For k factors and n center points, total experiments N = 2k(k − 1) + n	V	Efficiently estimates quadratic and interaction effects, building response surfaces with fewer runs than CCD; ideal when extreme axial points are costly or risky; robust for optimizing responses with moderate factor ranges	Tests only three levels—can only model up to second-order surface (quadratic); non-rotatable—variance depends on direction from center (poor prediction at extremes); less robust to missing data
Taguchi Arrays [49,118,119,120,121]	Categorical and continuous (level-based)	Taguchi orthogonal arrays (OA) test each parameter level across all others; robustness testing of system using signal-to-noise (S/N) ratios (i.e., deviation of response from desired value)—may have larger, smaller, or nominal S/N criteria; factor ranking by % contribution to response; Taguchi arrays are generalized Latin squares (handling more factors and levels)	OA is determined by number of factors and levels; runs depend on selected OA; L8 (eight runs), L9 (nine runs), etc. “L” denotes Latin square; number indicates total experiments Two-level Taguchi arrays—L4 (up to three factors), L8 (up to seven factors), L12 (up to 11 factors), L16 (up to 15 factors) Three-level Taguchi arrays—L9 (up to four factors), L18 (mixed: one two-level + up to seven three-level factors), L27 (up to 13 factors)	III	Reduces variability of a system (process or product); identifies optimal factor level at which variability in response due to noise (expensive or hard-to-control factors) is minimum	Good for robust parameter identification but less predictive; assumes negligible factor interactions—may produce inaccurate models; less flexible for full optimization
Latin Square Design (LSD) [122,123,124,125,126]	Categorical	Minimizes experimental error from two uncontrollable factors (i.e., operator and machine) through balanced design; simultaneous blocking in two directions (row and column)	Treatments arrange in a square array (appear once per row, once per column); treatment number = row blocks = column blocks	N/A (assumes no between-blocks or treatment-block interactions)	Comparing more than two treatments with two or even more nuisance factors in LSD extensions; more efficient than RCBD	Requires equal blocking/treatment levels; suitable for 5–10 treatments but laborious beyond that (Note: 2 × 2 Latin square designs not possible)
Mixture Design [127,128,129,130]	Continuous	Optimize blends (best mix of ingredients) in multi-component systems; all factors should measure in common unit (i.e., mass or volume)	Binary, ternary, and quaternary designs use two, three, and four components (forming a line, triangle, and tetrahedron in one, two, and three dimensions, respectively)	III, IV, or V	Scaling up formulations from lab scale to industrial scale (when formulation components change proportionally)	Not independent factor levels (must sum to a fixed total or stay within range); model looks at individual and simple combined effects (synergistic or antagonistic) but no squared/cubic terms
Plackett–Burman Design (PBD) [131,132,133,134,135]	Categorical (two levels) and continuous (two levels)	Economically screens k = N − 1 factors in N runs	Can test up to 11, 19, and 27 factors in 12, 20, and 28 runs, respectively (runs multiples of four, unlike powers of two in fractional factorials)	III (screens main effects only, ignores all interactions)	Quick main effects screening with very few runs, assuming no interactions	Only estimates large main effects (linear terms only); cannot estimate 2FI (main effects aliased with 2FI); no built-in replication to estimate pure error
Definitive Screening Design (DSD) [136,137,138,139,140,141]	Categorical (two levels) and continuous (thre levels)	Tests 4–30 numeric (3 levels) and up to 4 categorical (2 levels) factors; detects main effects, some 2FI and quadratic effects	For even k factors: 2k + 1 runs (i.e., eight factors in 17 runs) For odd k factors: 2k + 3 runs (i.e., seven factors in 17 runs) Center points added to estimate curvature, slightly increase total runs	IV (unaliased main effects, two-factor interaction partly confounded with square terms)	Screens many factors (>6) efficiently, detects nonlinearity	Not suitable if some factor combinations are impossible (assumes each factor can be changed freely); cubic terms confounded
Randomized Complete Block Design (RCBD) [142,143,144]	Treatment factors (categorical or discrete-level continuous); blocking factors (categorical)	All treatment levels are randomized within every homogeneous block, controlling one nuisance factor (block effect) * Called as randomized block designs (RBD) when some treatment-block combinations are missing (or incomplete)	Runs = N = t x b where t = treatment number (i.e., three different drug concentrations) and b = homogenous block number (i.e., four different batches of raw material or four different days)	N/A (controls known variation using blocks)	Agriculture/field trials to control variability from known nuisance factors (soil variation, shade, drainage, etc.) using blocking	Suitable for few treatments only (homogeneous blocks); controls only one known source of variation (other uncontrolled variations add to error)
Balanced Incomplete Block Designs (BIBD) [145,146,147,148,149]	Treatment factors (categorical or discrete-level continuous); blocking factors (categorical)	Incomplete blocks (fewer than total number of treatments per block) are balanced (equal repeats of each treatment and each treatment pair across design)	Two conditions must be satisfied: tr=bk and λ(t−1)= r(k−1) where t = treatment no., r = no. of treatment replications, b = block no., k = block size (no. of experimental units per block), and λ = no. of times every treatment pair occurs together in the same block	N/A; treatments are partially confounded with blocks (as not all treatments appear in every block)	Suitable for many treatments when blocks cannot fit all treatments	Cannot compare all treatment pairs directly (as each block only holds a subset of treatments); sensitive to missing data; unsuitable if treatment-block interactions expected
Optimal Design [90,93,95,112,150,151]	Best designs for mixed continuous and categorical factor types	Flexibility with factor types (continuous, numeric, or categoric) and levels (two, three, four, or more) D-optimal: minimizes variance of parameter estimate (coefficient) Optimal: minimizes average prediction variance Optimal: minimizes average variance of individual parameter estimates E-optimal: minimizes the worst parameter variance G-optimal: minimizes the worst prediction variance	Test 1–30 numeric, 1–10 categorical factors	III, IV, or V	When standard designs cannot accommodate experimental adjustments; for irregular design space	“Model-dependent” design (captures interactions/curvature only if specified—requiring up-front interaction terms and three factor levels); flawed conclusions if wrong model selected; lack of replicates and reduced robustness; possibility of local optimum; may require extreme (impractical) factor settings

**Table 3:** Strategic framework for experimental integrity: DOE pitfalls and statistical safeguards.

Sr.	DOE Pitfall	How to Avoid (Statistical Safeguards)
1.	Unclear objectives or critical responses	Define clear goals and CQAs up front to ensure relevant data and interpretable models [48,49,161].
2.	Factor misclassification	Properly distinguish between categorical (types) and continuous (numeric) factors during setup to avoid distorted analyses and misleading surfaces [90].
3.	Poor factor range selection	Select realistic factor bounds for effect size detection: levels should be far enough apart to see a true signal (and avoid catching noise), but close enough to avoid extreme, unmanageable nonlinearity [56,162].
4.	Wrong design selection	Use screening designs (i.e., Taguchi design or PBD) for important factor identification; switch to RSM (i.e., BBD/CCD) for optimization [67,72].
5.	Neglecting noise control	Use randomization of run order for detection of long-term variability and noise distribution [72], blocking for batch effects (block nuisances) and replication for error estimation.
6.	Skipping center point replicates	Include three to five center points to estimate pure error and test curvature and lack of fit [72,90].
7.	Overreliance on high R^2^ alone	R^2^ always increases with added terms, risking overfitting [49,152]. Prioritize a non-significant lack of fit (*p* > 0.05) over a high R^2^ to ensure the model actually fits the data shape. Check PRESS and %CV diagnostics and ensure adjusted–predicted R^2^ gap < 0.2.
8.	Ignoring interaction and quadratic effects	Include AB (interaction) and A^2^ (quadratic) terms to capture synergistic effects and nonlinear behavior (curvature) in response, respectively [90].
9.	Model overfitting (i.e., unnecessary cubic terms)	Select the highest-order polynomial with significant and non-aliased terms. Apply sequential model selection (mean → linear → 2FI → quadratic) using *p* < 0.05 or AIC for parsimony (parsimony principle favors the simplest adequate model, avoiding unnecessary complexity) [152,153].
10.	Breaking hierarchy	Always retain linear precursors when quadratic terms are included to preserve model validity (i.e., keep A if A^2^ or AB is in the model) [49].
11.	Overlooking non-constant variance and Box–Cox transformations	Box–Cox transformation stabilizes variance (heteroscedasticity), normalizes skewed data for ANOVA, and simplifies complex interaction models [54,159,163]. If 95% CI for λ includes 1, no transformation needed; otherwise, use square root or log alternatives. Note: Box–Cox cannot handle negative values; non-integer powers yield complex numbers.
12.	Risky extrapolation	Interpolate only within tested factor region/boundaries; predictions outside the design space are statistically unreliable [152].
13.	No confirmation runs at predicted optima	Always confirm model predictions experimentally by performing three confirmation replicates at the predicted “optimum” point to verify the model’s accuracy [72].

**Table 4:** Graphical tools for model interpretation in Design-Expert^®^ software.

Category	Plot	Purpose and Interpretation
DIAGNOSTIC PLOTS (Model Validation)	Box–Cox Plot [54,163,167]	Box–Cox plot identifies the optimal response transformation (λ) at the minimum of the curve from scaled log (SS residuals). If the 95% confidence interval around λ includes 1, no transformation is needed; otherwise, Design-Expert^®^ provides options such as square root, inverse, or logarithmic transformations to stabilize variance (heteroscedasticity).
	Normal Probability of Residuals [49,157,168]	Verifies if residuals (not the raw data) follow a normal distribution (should form a straight line)
	Residual vs. Predicted [49,54,157,168]	Checks for assumption of constant variance (homoscedasticity) of residuals; a random scatter confirms the model is unbiased.
	Residual vs. Run [49,157]	Detects time-related effects (or lurking variables). Random scatter indicates their absence, while trends suggest time-related factors, which can be addressed through blocking and randomization.
	Predicted vs. Actual [49,167]	Directly visualizes how well the model predicts data compared to observed results (goodness of fit).
INFLUENCE PLOTS (Point Precision)	Leverage Plot [54]	Evaluates the capacity of a design point (based on its position in design space) to affect overall model fit (predictions).
	Cook’s Distance [54,169]	Quantifies influence of individual point/observation on model fit (by measuring the overall change in model coefficients) when a specific point is removed.
	DFFITS (Difference in Fits) [169]	It measures change in predicted values (fitted values) when an observation (run) is excluded.
	DFBETAS (Difference in Coefficients or Betas) [54,169,170]	Deletion diagnostic that tracks change in regression coefficient when an individual point/run is excluded.
MODEL PLOTS (Data Visualization)	Perturbation Plot [54,167]	Displays effect of individual factor on response while holding all other factors constant at a reference point (Design-Expert defaults reference points to mid-factor levels, coded 0); illustrates single-factor sensitivity similar to OFAT experiments; steeper slopes indicate higher factor impact; curvature indicates quadratic effects.
	Contour Plot (2D) [49,54,90]	2D representations showing response behaviour as contour lines for two factors, with other factors held constant via color coding (blue for low and red for high response).
	3D Surface Plot [49,54,90]	Provides a 3D projection of the 2D contour plot; visualizes response surface curvature and stationary points (peaks, valleys, or saddle points) for response optimization.

**Table 5:** Factors and levels for Taguchi L_8_ orthogonal array.

Run	A: Drug Amount (mg)	B: Aqueous Vol (mL)	C: Infusion Speed (mL/min)	D: Stirring Speed (RPM)
1	5	4	2	300
2	5	4	4	500
3	5	6	2	300
4	5	6	4	500
5	15	4	2	500
6	15	4	4	300
7	15	6	2	500
8	15	6	4	300

**Table 6:** Summary of ANOVA for all responses for screening phase (L_8_ design).

Source		A: Drug Amount	B: Aqueous Volume	C: Infusion Speed	D: Stirring Speed	Residual	Total
Response 1: Entrapment Efficiency (EE%)	SS	968.00	0.32	2.00	0.18	0.34	970.84
	MS	968.00	0.32	2.00	0.18	0.34	970.84
	F-value	8541.18	2.82	17.65	1.59	—	—
	*p*-value	<0.0001	0.1915	0.0246	0.2967	—	—
	Contr.	99.71%	0.033%	0.206%	0.019%	0.035%	100%
Response 2: NP Size (nm)	SS	1.526 × 10^10^	2.348 × 10^9^	5.113 × 10^6^	3.816 × 10^7^	1.621E × 10^6^	1.765 × 10^10^
	MS	1.526 × 10^10^	2.348 × 10^9^	5.113 × 10^6^	3.816 × 10^7^	1.621E × 10^6^	1.765 × 10^10^
	F-Value	28,226.09	4344.76	9.46	70.60	—	—
	*p*-Value	<0.0001	<0.0001	0.0543	0.0035	—	—
	Contr.	86.44%	13.31%	0.029%	0.216%	0.009%	100%
Response 3: Zeta Potential (mV)	SS	59.41	0.6050	0.1800	0.1250	0.4050	60.72
	MS	59.41	0.6050	0.1800	0.1250	0.4050	60.72
	F-Value	440.04	4.48	1.33	0.9259	—	—
	*p*-Value	0.0002	0.1245	0.3318	0.4069	—	—
	Contr.	97.83%	0.996%	0.296%	0.206%	0.667%	100%
DF		1	1	1	1	3	7

SS, sum of squares; MS, mean square; Contr., percentage contribution; DF, degree of freedom

**Table 7:** Combined Taguchi response summary (means and S/N ratios).

Response	Factor	Level 1 (Mean)	Level 2 (Mean)	Delta (Mean)	Rank (Mean)	Level 1 (S/N)	Level 2 (S/N)	Delta (S/N)	Rank (S/N)
Entrapment Efficiency (EE%) (larger is better)	A: Drug Amount	38.50	16.50	22.00	1	31.71	24.34	7.37	1
	B: Aqueous Volume	27.70	27.30	0.40	3	28.09	27.96	0.13	4
	C: Infusion Speed	27.00	28.00	1.00	2	27.81	28.24	0.42	2
	D: Stirring Speed	27.65	27.35	0.30	4	28.16	27.89	0.26	3
NP Size (nm) (smaller is better)	A: Drug Amount	141.9	172.6	30.7	1	−43.03	−44.74	1.70	1
	B: Aqueous Volume	151.2	163.4	12.2	2	−43.54	−44.23	0.69	2
	C: Infusion Speed	157.4	157.1	0.4	4	−43.89	−43.88	0.01	4
	D: Stirring Speed	156.5	158.0	1.5	3	−43.84	−43.92	0.08	3
Zeta Potential (mV) (larger is better)	A: Drug Amount	−19.88	−14.43	5.45	1	25.97	23.18	2.79	1
	B: Aqueous Volume	−17.43	−16.88	0.55	2	24.73	24.42	0.31	2
	C: Infusion Speed	−17.00	−17.30	0.30	3	24.48	24.66	0.18	3
	D: Stirring Speed	−17.28	−17.03	0.25	4	24.65	24.49	0.15	4

**Table 10:** Fit statistics for measured responses, EE%, NP size, and zeta potential.

Response	Entrapment Efficiency (EE%)			NP Size (nm)			Zeta Pot. (mV)
Model	Mean	Linear	2FI	Mean	Linear	2FI	Mean
R^2^	0.000	0.734	0.973	0.000	0.483	0.803	0.000
Adjusted R^2^	0.000	0.674	0.962	0.000	0.369	0.728	0.000
Predicted R^2^	−0.19	0.421	0.947	−0.19	−0.015	0.600	−0.190
Adequate Precision	NA ⁽^1^⁾	9.7	30.3	NA⁽^1^⁾	5.7	10.6	NA⁽^1^⁾
CV %	75.3	42.9	14.6	8.4	6.7	4.4	15.3

Coefficient of determination (R^2^) is a measure of total variability explained by the chosen model. Adjusted R^2^ assesses model fit accounting for insignificant predictors, while predicted R^2^ evaluates predictive reliability for new data. ^(1)^ Adequate Precision is NA for mean models because constant predictions yield the signal range zero and the ratio mathematically incalculable.

**Table 11:** Model comparison statistics for responses, EE%, NP size, and zeta potential.

Response	Entrapment Efficiency (EE%)			NP Size (nm)			Zeta Pot. (mV)
*Model/Statistics*	*Mean*	*Linear*	*2FI*	*Mean*	*Linear*	*2FI*	*Mean*
PRESS	1.11 × 10^−6^	5.38 × 10^−7^	4.92 × 10^−8^	2025	1728	681	90
−2 LL	−162.43	−178	−206	94	86	74	56
BIC	−160	−171	−196	96	93	84	59
AIC	−160	−169	−192	96	95	88	59

PRESS, predicted residual error sum of squares; BIC, Bayesian information criteria; AIC, Akaike’s information criteria; −2LL, −2 log likelihood.

**Table 12:** Results of ANOVA (best-fit 2FI model) for %EE.

Response: EE Transform: Power, Lambda: −2.68, Constant: 0						
Source	Sum of Squares (SS)	df	Mean Square (MS)	F-value	p-value	
Model	9.037 × 10^−7^	3	3.012 × 10^−7^	94.29	<0.0001	significant
A: Drug Amount	4.904 × 10^−7^	1	4.904 × 10^−7^	153.51	<0.0001	
B: Aqueous Volume	1.913 × 10^−7^	1	1.913 × 10^−7^	59.87	<0.0001	
AB	2.220 × 10^−7^	1	2.220 × 10^−7^	69.49	<0.0001	
Residual	2.556 × 10^−8^	8	3.195 × 10^−9^			
Lack of Fit	8.051 × 10^−9^	5	1.610 × 10^−9^	0.2759	0.9002	not significant
Pure Error	1.751 × 10^−8^	3	5.836 × 10^−9^			
Cor Total	9.293 × 10^−7^	11				

Cor Total, corrected total (indicates sum of squares (SS) corrected for the mean, not zero baseline).

**Table 13:** Results of ANOVA (best-fit 2FI model) for NP size.

Response: Size Transform: None						
*Source*	*Sum of Squares (SS)*	*Df*	*Mean Square (MS)*	*F-value*	*p-value*	
Model	1365.40	3	455.13	10.84	0.0034	significant
A: Drug Amount	286.39	1	286.39	6.82	0.0311	
B: Aqueous Volume	536.12	1	536.12	12.76	0.0073	
AB	542.89	1	542.89	12.92	0.0070	
Residual	336.03	8	42.00			
Lack of Fit	102.97	5	20.59	0.2651	0.9065	not significant
Pure Error	233.07	3	77.69			
Cor Total	1701.44	11				

**Table 14:** Results of ANOVA (best-fit mean model) for NP zeta potential.

Response: Zeta Potential Transform: None						
*Source*	*Sum Of Squares (SS)*	*df*	*Mean Square (MS)*	*F-Value*	*p-Value*	
Model	0.0000	0		-	-	Significant
Residual	75.70	11	6.88			
Lack Of Fit	49.35	8	6.17	0.7024	0.6942	Not Significant
Pure Error	26.35	3	8.78			
Cor Total	75.70	11				

**Table 15:** Numerical optimization solutions for model verification.

No.	Drug Amount (mg)	Aqueous Volume (ml)	EE (%)	NP Size (nm)	Zeta Potential	Desirability	
**1**	**5.0**	**4.0**	**47.6**	**133**	**−17.2**	**0.961**	**Selected**
2	5.0	4.7	31.2	148	−17.2	0.903	
3	5.0	4.8	30.5	149	−17.2	0.898	
4	5.0	5.9	24.1	170	−17.2	0.814	
5	5.0	6.0	23.6	173	−17.2	0.803	
6	5.0	6.0	23.6	173	−17.2	0.802	
7	10.6	6.0	23.0	153	−17.2	0.788	
8	11.2	6.0	23.0	151	−17.2	0.787	
9	12.3	6.0	22.9	148	−17.2	0.784	

**Table 16:** Optimized formulation.

Response	Predicted Mean	Observed Mean	Standard Deviation	% Accuracy	95% PI * Lower	95% PI * Upper
EE (%)	47.6	41.2	13.3	87	21.4	73.8
NP Size (nm)	133	124	6.0	93	121.2	144.8
Zeta Potential (mV)	−17	−15	3.0	87	−22.9	−11.1

* Assuming normal distribution, approximate 95% PI = predicted mean ± 1.96 × SD.

## Data Availability

Data are contained within the article. The practical case study on poly(lactic-co-glycolic acid) (PLGA) nanoparticles was conducted, analyzed, and reported entirely by the authors herein.

## Glossary of DOE Terminology

**Terminology**	**Definition**
Bias	Bias is any systematic trend or deviation from truth in data collection, analysis, interpretation, or publication, leading to erroneous conclusions.
Blocking	Blocking involves grouping similar experimental units into blocks, aiming to increase precision by minimizing the impact of known nuisance variables (e.g., raw material batch).
Categorical factor	Qualitative variable that categorizes data into distinct groups (e.g., polymer type).
Confounding (or aliasing)	Confounding occurs when the effects of two or more factors are merged into a single observed effect, losing the orthogonality and independence of variables.
Continuous factors	Continuous factors encompass any real value within a range (e.g., drug amounts).
Controlled variables	Variables that are controlled or held constant during the experiment (e.g., centrifuge speed).
Degrees of freedom (DF)	Degrees of freedom (DF) are the number of independent, fair comparisons possible in a dataset, after accounting for estimated parameters. Total experiment DF = no. of experiments − 1. Factor DF = levels − 1. Interaction DF = product of factor DFs. Error DF = total − model DF.
Design space	Design space is a multidimensional parameter space that defines the permissible operational ranges for input variables and process parameters to ensure consistent product quality.
Discrete factors	Discrete factors denote specific values counted as whole numbers (e.g., mixing speed of 400, 500 RPM).
Effect or main effect	Factor effect averaged across all levels of other independent variables. It represents the extent of change in response resulting from variations in factor level(s).
Experimental design	Experimental design is the systematic, controlled process of manipulating independent variables to maximize precision and draw valid conclusions about their effects on dependent responses.
Factor or independent variables	Input variable (experimental factor) that is systematically manipulated or controlled to assess its impact on the dependent variable or response (e.g., drug amount, solvent volume).
Factor level	Value of the factor that is used in the experiment (i.e., low, medium, and high).
Homoscedasticity	Homoscedasticity is the statistical assumption that the “variance of the residual error” remains constant across all levels of factors.
Interaction	Interaction arises when the impact of one factor varies depending on the level of another factor (s), signifying non-additive effects among factors.
Noise (or nuisance or lurking variables)	Noise (or lurking variables) are uncontrolled, often costly-to-manage factors—such as ambient humidity or raw material batches—that inflate experimental error and obscure the true effects of factors.
Numeric or quantitative factor	A numeric variable represents measurable quantities; it can be a continuous or discrete numeric variable.
Orthogonality	Orthogonality allows independent evaluation of factors or interactions in a model (uncorrelated variables); removal of one term does not alter the other parameter estimates or their respective sum of squares in the ANOVA (preferred but not essential with modern computational tools).
Randomization	Randomization assigns treatments/factors randomly (based on probability alone), averaging out uncontrolled variables and preventing bias.
Replication	Replication involves repeating an experiment or its parts under identical conditions to estimate pure experimental error and enhance the precision of effect estimates.
Residuals	Quantitative difference between the observed and the predicted response.
Resolution	Resolution (within a fractional factorial design) is a design’s ability to separate main effects from various multifactor interactions.
Response surface methodology (RSM)	RSM utilizes second-order designs and quadratic equations to map multi-factor relationships into 3D surfaces, enabling simultaneous optimization with fewer experimental runs.
Response (or dependent variable)	A response measures the outcome of interest, reflecting changes caused by the manipulation of experimental factors (e.g., EE% and NP size).
Robustness	Robustness confirms that minor, deliberate changes in experimental factors do not significantly impact the response (ensuring the process remains stable under real-world conditions).
Rotatable design	A rotatable design ensures that the variance of predicted response remains constant at all points equidistant from the design center, regardless of direction (ensuring uniform precision in every direction of the design space).
Runs	A run is a single experimental trial or test conducted (in a randomized sequence) at a specific combination of factor levels to measure the resulting response.
Screening designs	Preliminary design of experiment used to shortlist (screen) a couple of significant factors impacting the response.
Treatment	Unique combination of factor settings or levels.
